# Central nervous system targeted autoimmunity causes regional atrophy: a 9.4T MRI study of the EAE mouse model of Multiple Sclerosis

**DOI:** 10.1038/s41598-019-44682-6

**Published:** 2019-06-11

**Authors:** A. Max Hamilton, Nils D. Forkert, Runze Yang, Ying Wu, James A. Rogers, V. Wee Yong, Jeff F. Dunn

**Affiliations:** 1 Department of Radiology, Cumming School of Medicine, University of Calgary, Calgary, Alberta Canada; 2 Hotchkiss Brain Institute, Cumming School of Medicine, University of Calgary, Calgary, Alberta Canada; 3 Department of Clinical Neurosciences, Cumming School of Medicine, University of Calgary, Calgary, Alberta Canada

**Keywords:** Multiple sclerosis, Cell death in the nervous system, Magnetic resonance imaging

## Abstract

Atrophy has become a clinically relevant marker of progressive neurodegeneration in multiple sclerosis (MS). To better understand atrophy, mouse models that feature atrophy along with other aspects of MS are needed. The experimental autoimmune encephalomyelitis (EAE) mouse model of MS was used to determine the extent of atrophy in a model of inflammation-associated central nervous system pathology. High-resolution magnetic resonance imaging (MRI) and atlas-based volumetric analysis were performed to measure brain regional volumes in EAE mice. EAE brains were larger at peak clinical disease (days 14–16) compared to controls, with affected regions including the cerebellum, hippocampus, and corpus callosum. Following peak clinical disease, EAE mice exhibited significant loss of volume at chronic long-term disease duration (day 66+). Atrophy was identified in both white and grey matter regions including the cerebral cortex, cerebellum, hippocampus, corpus callosum, basal forebrain, midbrain, optic tract, and colliculus. Histological analysis of the atrophied cortex, cerebellum, and hippocampus showed demyelination, and axonal/neuronal loss. We hypothesize this atrophy could be a result of inflammatory associated neurodegenerative processes, which may also be involved in MS. Using MRI and atlas-based volumetrics, EAE has the potential to be a test bed for treatments aimed at reducing progressive neurological deterioration in MS.

## Introduction

Multiple sclerosis (MS) has generally been thought of as a white matter (WM) disease characterized by neuroinflammation and demyelination of the brain and spinal cord. Current evidence, however, indicates that neurodegenerative processes including axonal degeneration, and the loss of cells including neurons and oligodendrocytes, play an important role alongside inflammation and affects both white and grey matter (GM) in the brain^1–3^. The accumulation of these neurodegenerative processes results in a loss of brain volume known as atrophy^4–6^. Atrophy occurs at the earliest stages of the disease^7^, and plays a prominent role in progressive MS^8^. Atrophy has been associated with disease progression^9^, physical symptoms^10^, and cognitive impairment^11^, making atrophy one of the most clinically relevant markers of progressive disability in MS^12^.

With the importance of neurodegeneration and atrophy, understanding what drives these processes is critical to identifying therapeutic targets and stopping disease progression. Inflammation for example has been associated with regions of axonal damage and transections in relapse remitting MS (RRMS)^13^. This is further supported by studies having found correlations between the rate of brain atrophy and the number of active T1 lesions^14^, and early MS lesion accumulation with atrophy years later^15^. Finally, inflammation is still present during secondary progressive MS (SPMS), and is associated with active demyelination and neurodegeneration^16^ suggesting it may still be associated with atrophy in this phase of the disease.

Despite this evidence, anti-inflammatory drugs have had little effect at reducing atrophy, and have not been affective at treating clinical disability in progressive forms of the disease when atrophy is most prominent. As a result, it is unclear what the relationship is between inflammation and atrophy.

The experimental autoimmune encephalomyelitis (EAE) mouse model could be a good candidate for studying such a linkage as it is widely used to study neuroinflammation. In EAE, researchers have found evidence of inflammation, demyelination, or neurodegeneration in a number of regions including the hippocampus^17,18^, striatum^19,20^, cerebellum^21^, corpus callosum^22^, and the cerebral cortex^23–25^. The cellular degeneration observed in these studies make it possible that there is atrophy. A study using MRI^24^ detected a significant reduction in the volumes of the cerebellum, and cerebral cortex, *in-vivo*, in the EAE model at long-term disease duration. This suggests that the EAE model may, in addition to its modeling neuroinflammation, be a model of atrophy.

While promising, the regions studied were limited and so it is unknown how widespread atrophy is in EAE, if WM atrophy occurs alongside GM, and if the regions affected are similar to MS. The aim of this study was to determine how brain morphology changes in a mouse model of neuroinflammation, and whether an autoimmune response can result in atrophy affecting similar regions as in MS. High-resolution MRI, and atlas-based regional volumetrics were used in this study to measure the volumes of anatomical regions in the brains of living EAE mice.

## Materials and Methods

### Mice

Female C57BL/6 mice (Charles River Laboratories, 8–10 weeks) were housed on a 12 hr light/dark cycle. Food and water were available *ad libitum*. All animal procedures were approved by the Animal Care Committee at the University of Calgary and were in accordance with the guidelines established by the Canadian Council on Animal Care.

### EAE induction

Mice were allowed to acclimatize to the animal house facility for two weeks before EAE induction. At 10–12 weeks old, mice were immunized as described previously^26^. In brief, mice were immunized with 50 µg of myelin oligodendrocyte glycoprotein (MOG)_35–55_ to trigger an autoimmune response. MOG was mixed with Complete Freund’s Adjuvant (CFA) comprised of 10 mg/ml of heat inactivated *mycobacterium tuberculosis* emulsified in Freund’s Adjuvant, which helped boost the immune system facilitating disease induction. EAE induction was performed via two 50μl subcutaneous injections into each side flank of the mice while they were under light ketamine/xylazine anesthesia. Mice were injected intraperitoneally with 300 ng/250 μl of pertussis toxin (PTX) immediately and two days after MOG immunization. To assess motor disability and disease severity, mice were scored on a 15-point grading scale with each limb and the tail scored separately^27^. Controls included a naïve group, which received no injections, and CFA mice, which received all injections including CFA and PTX, but no MOG. All mouse groups (Naive, CFA, and EAE) were subjected to the same handling and behavior scoring and were weighed following scoring. All mice were imaged at 66 days post-induction as part of a “Long term” comparison (n = 9 Naïve, 13 CFA, 27 EAE). A subset of these mice imaged at long-term (n = 9 Naïve, 7 CFA, 12 EAE) were also imaged at days 14–16, as part of a comparison at “Peak” clinical disease severity. This time point was selected as our EAE mice will on average reach their highest disability scores 14–16 days post disease induction. Mice that showed large discrepancies with this timing were excluded from the study. The mice imaged at both “Peak” and “Long-term,” also enabled “Peak to Long-term time-course” comparisons.

### MRI acquisition

Mice were anesthetized with 1–2% isoflurane during imaging. Imaging was conducted using a 9.4T Bruker MRI with an Avance console, paravision 5.1, and a helium cooled Bruker Cryoprobe. Mice were imaged using a gradient echo sequence (matrix size = 512 × 512; field of view = 19.2 mm × 19.2 mm; slice number = 60; repetition time (TR) = 1500 ms; echo time (TE) = 6.5 ms; number of averages = 3; resolution = 37.5 μm × 37.5 μm × 250 μm; flip angle = 60°).

### Image processing

**Figure 1:**
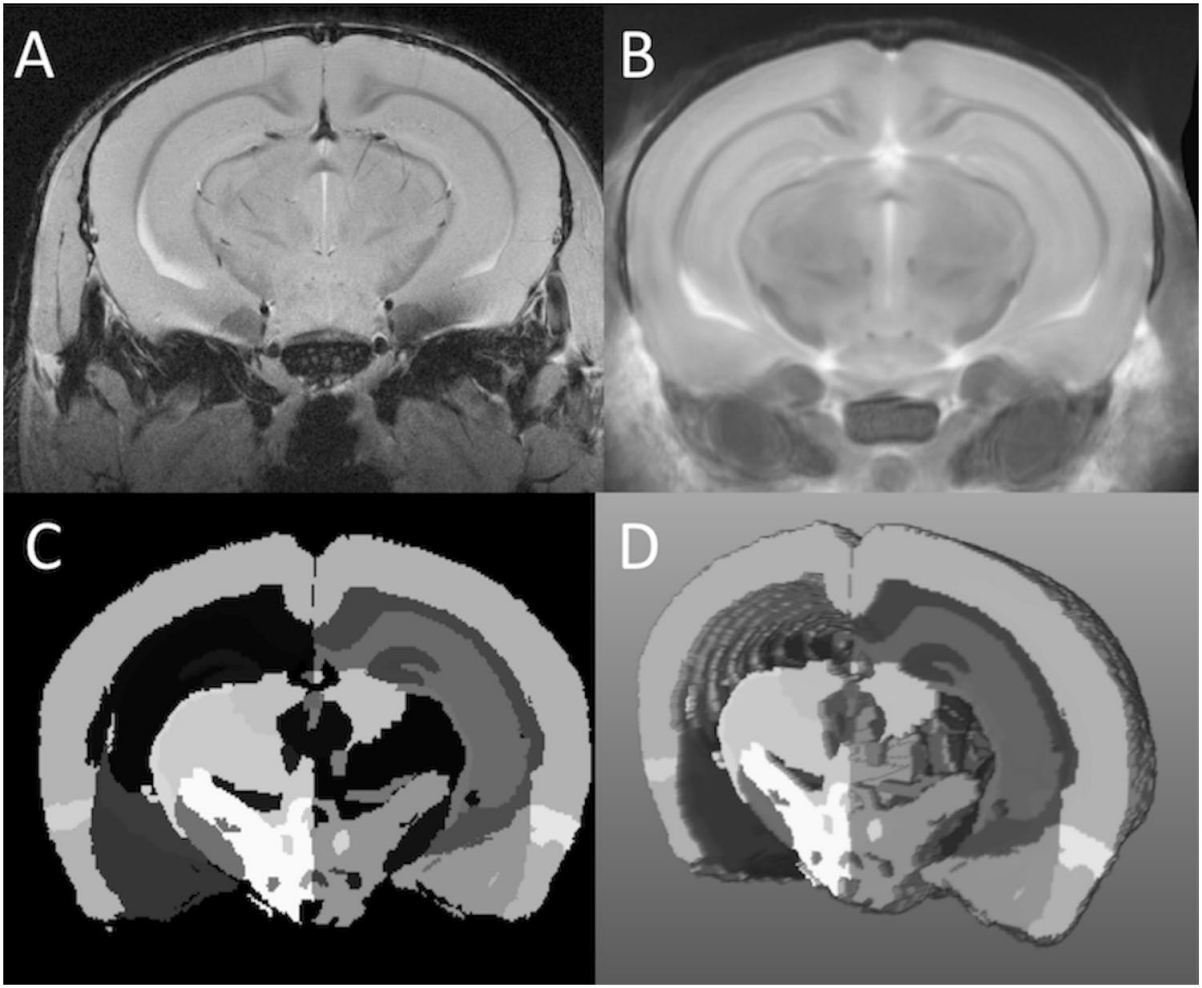
Atlas-based regional volumetrics. (**A**) Gradient-echo MRI image of an EAE mouse brain 66 days post induction. Signal in-homogeneities caused by signal drop off from the surface coil have been corrected with the N3 algorithm. (**B**) The mouse brain atlas has undergone image registration to align with the EAE mouse brain seen in A. (**C**) EAE mouse brain segmented into the 62 measured structures. (**D**) 3D representation of the segmented EAE mouse brain.

Image intensity was normalized using the N3 algorithm^28^, to correct for signal drop-off associated with surface coils (Fig. 1A). A previously generated mouse atlas that had been segmented into 62 structures was used for image registration (Fig. 1B)^29^. The atlas was registered non-linearly to individual images using the software Niftyreg (Fig. 1C)^30^. After image registration, the corresponding non-linear transformation was used to transform the 62 atlas brain regions to each individual case using a nearest-neighbor interpolation (Fig. 1D). Following segmentation, images were verified using MeVisLab (version 3.1.1) to ensure the atlas was properly registered to the original images. Segmented images were compared alongside the original MRI images with a synchronized cursor to determine if boundaries of the brain and major anatomical regions were aligned. The transformed atlas regions were then used for volumetric analysis of each brain structure.

### Tissue preparation and staining

Mice were perfusion fixed 66 days post induction using phosphate buffered saline (PBS) followed by 4% paraformaldehyde (PFA). Mouse brains were removed and placed in 4% PFA overnight before being placed in a 30% sucrose solution. Brains were frozen sectioned into 20 μm thick slices for staining. For each stain, three slices 150μm apart were processed per animal. The following antibodies were used: myelin basic protein (MBP) (Abcam Cat# ab40390) for myelin, SMI-312 (BioLegend Cat# 837904) for axons, and anti-neuronal specific nuclear protein (NeuN) (Abcam: Abcam Cat# ab177487) for neurons. Secondary stains included Alexa Fluor 488 and 594 (Jackson ImmunoResearch Labs Cat# 711-545-152; Cat# 711-585-152; Cat# 715-585-150). Tissue was blocked for 1 hour with 10% horse serum, 1% bovine serum albumin (BSA), 1% Triton-X100, 0.1% cold fish skin gelatin (CFSG), 0.05% Tween-20 in PBS. Primary antibodies were diluted and incubated overnight with 5% horse serum, 1% BSA, 0.1% CFSG, and 1% Triton-X100 in PBS. Secondary antibodies were diluted and incubated for 1 hour with 1% BSA, 0.1% CFSG, and 1% Triton-X100 in PBS.

### Stereology

Cortex, hippocampus, and cerebellar sections were analyzed (n = 5 Naïve, 5 CFA, 10 EAE). Cell counting was performed using stereology^31^. Quantification of NeuN stained neurons was performed using a computer interfaced with an Olympus BX61-DSU microscope with a motorized stage, running StereoInvestigator software (version 11, MicrobrightField). Three slices 150 um apart were used for each animal, for each structure of interest. Regions of interest were traced at low magnification (4x magnification, NA 0.16). Counting was performed using random counting grids (35 μm × 35 μm cortex, 10 μm × 10 μm hippocampus, and cerebellum) at 40x magnification (NA 0.45). Counting sites per section ranged from 25–30 for the cortex and hippocampus, and 40–50 for the cerebellum.

Myelin and axon levels (n = 5 Naïve, 5 CFA, 5 EAE) were quantified with percent area of immunoreactivity measurements using Fiji (ImageJ) version 1.51b (Mac version of NIH Image), downloaded from https://imagej.net/Fiji/Downloads. Three slices 150 um apart were used for each animal, for each structure of interest. For each tissue slice, three adjacent images were taken of the structure of interest for a total of nine images per structure. To avoid experimenter bias, auto-adjustment of brightness and contrast, as well as threshold of staining signal were used.

### Statistics

All statistical analyses were conducted using SPSS (version 24, SPSS Inc., Chicago, IL). To compare structural volumes between groups, a one-way analysis of covariance (ANCOVA) was conducted. To control for variance in brain size between animals, body weight was used as a covariate similar to previous studies^32,33^. This is because body size generally scales with brain size, such that a larger adult mouse would typically have a larger brain^34,35^. While previous studies controlled for body weight at the time of imaging, we controlled for body weight prior to EAE induction when animals were at adult size for brain volume, skull size, and body weight. EAE disease significantly reduces body weight with clinical disease severity and we do not expect that weight loss should directly result in brain atrophy^24^. Therefore, body weight during EAE disease would no longer reflect normal adult body size and be an appropriate control for normal adult brain size variance. We included the uncorrected volumes for the reader’s interest. To compare time-course changes in volume from peak clinical disease to long-term, relative changes in volume were calculated from the uncorrected peak and long-term data. Statistical comparisons of the uncorrected peak clinical disease, and long-term data, as well as the time course data, were performed using an ANOVA test. ANOVA/ANCOVA tests are sensitive to unequal variances which can be a problem when group sizes are significantly different. In these instances, a F test for heteroscedasticity was performed to ensure equal variances. To account for increased chance of type I error caused by multiple statistical comparisons, the false discovery method (FDR) was used^36^. A 10% FDR was selected as the cutoff for significance as this value has been used in previous volumetric studies^37,38^ and simulation studies^39^. p values corrected with the FDR method are presented as q.

At long-term, the EAE group was compared to the Naïve and CFA groups as a whole in each analysis and then divided into two groups based on long-term clinical disease scores. Long-term clinical disease scores were determined by summing up each daily clinical disease measurement from day 30 to 66 per mouse. This time point was chosen as long-term as clinical disease scores had plateaued and remained relatively constant following day 30. The cumulative clinical score represents the cumulative burden of disease of individual mice during the long-term phase of the disease. Cumulative long-term disease scores were then averaged amongst EAE mice, and mice with above average scores were grouped together as high score EAE while mice with below average scores were grouped together as low score EAE. Correlations were determined between long-term disease score and brain volume using a linear regression analysis. The strength of relationships were defined as 0.00–0.19 “very weak”, 0.20–0.39 “weak”, 0.40–0.59 “moderate”, 0.60–0.79 “strong”, and 0.80–1.0 “very strong”.

Statistical significance for immunohistochemistry and stereology data were determined using an ANOVA test with a Tukey post-hoc.

## Results

### EAE disease course

**Figure 2:**
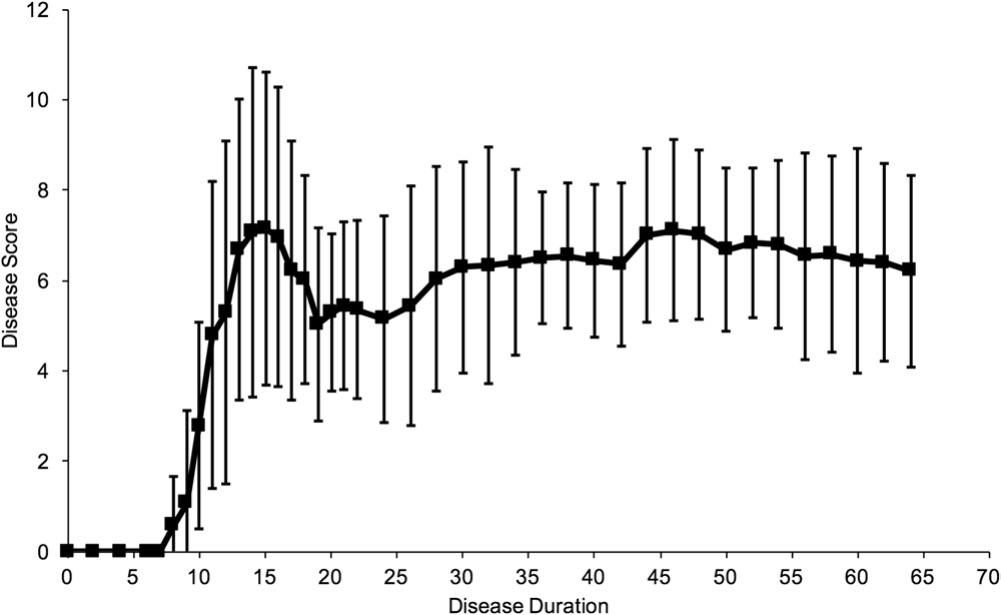
EAE Disease Course. Mean daily disease score and body weight of EAE mice (n = 27) over time following immunization. Error bars represent standard deviation.

Clinical signs manifested initially by paresis and then paralysis of the tail began to develop 8 days post immunization. These progressed to paresis/paralysis of the hind limbs, which then ascended to involve the fore limbs. Peak clinical disease severity occurred between days 14–16. Following peak disease, there was partial recovery of limb function followed by clinical disease plateauing around day 30. This long-term chronic paresis continued until the experimental endpoint (Fig. 2).

### EAE mice at peak clinical disease have increased brain volume compared to Naïve and CFA mice

**Figure 3:**
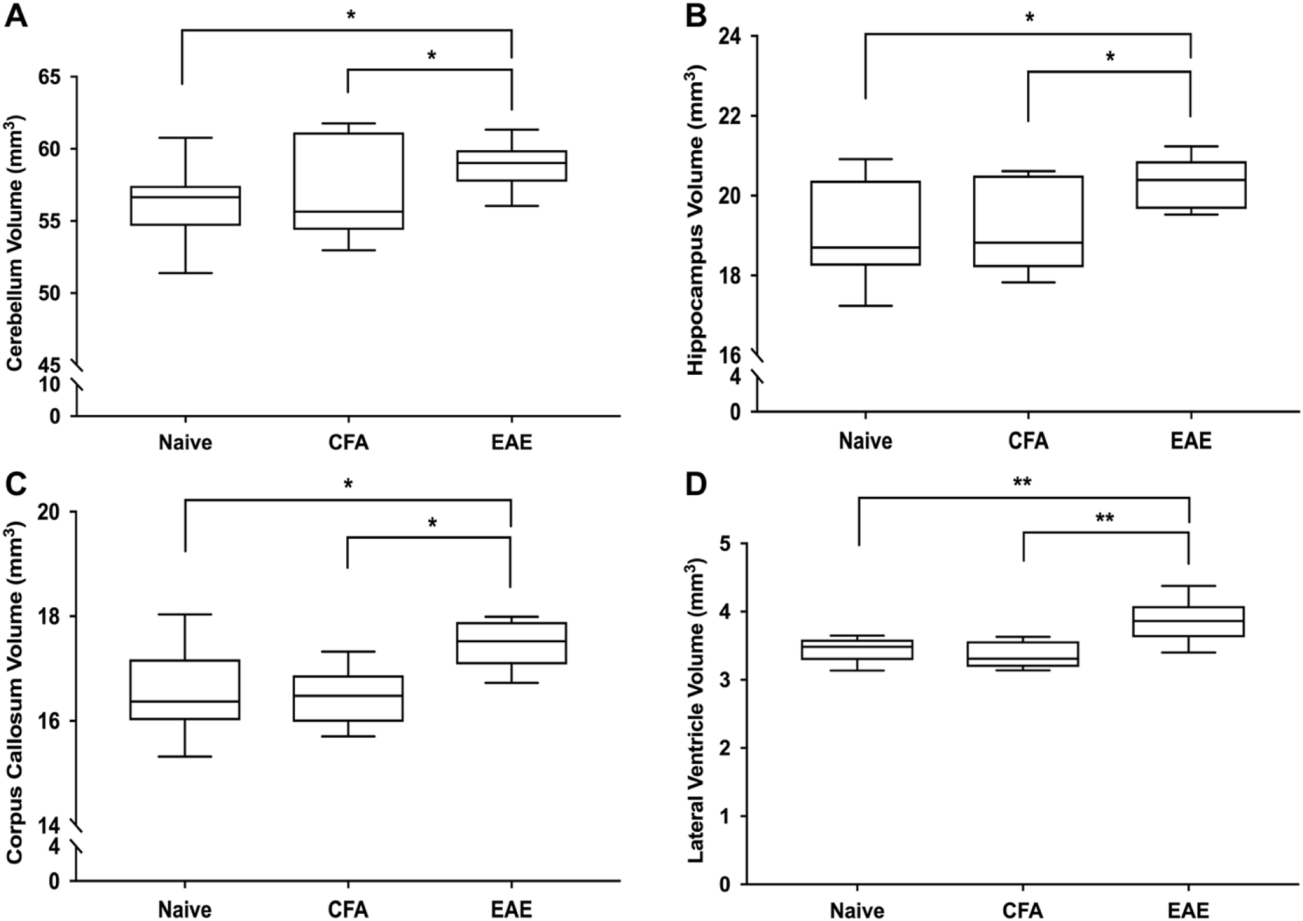
EAE mice as peak disease (days 14–16) have significantly larger brains. (**A**) Cerebellum volume (cerebellar cortex + arbor vita of cerebellum + cerebellar peduncle: inferior, middle, superior) in Naive, CFA, and EAE, mice. (**B**) Hippocampal volume. (**C**) Corpus callosal volume. (**D**) Lateral ventricle volume. All volumes are adjusted based on weight using an analysis of covariance (ANCOVA). n = 9 Naïve, 7 CFA, 12 EAE. *q < 0.05; **q < 0.01 ***q < 0.001. q values represent p values corrected for body weight and multiple comparisons via the false discovery rate (FDR) method.

After correcting for body weight, we found that EAE mice imaged at peak clinical disease had significantly larger brain sizes compared to the CFA or Naïve controls (422 ± 9 mm^3^, 407 ± 18 mm^3^, and 405 ± 15 mm^3^, respectively (mean ± SD); F_(3,22)_ = 4.86, q = 0.042, ANCOVA). Overall, 25 of the 62 structures had significantly larger volumes in EAE mice compared to CFA and Naïve controls (complete dataset in Supplementary Tables S1–4). The 24 structures were comprised of 10 GM and 11 WM regions as well as the ventricles. Structures with the largest differences included the cerebellum, hippocampus, corpus callosum, and the lateral ventricles (Fig. 3). Other affected regions included the fimbria, basal forebrain, periaqueductal grey, cerebral aqueduct, third ventricle, pons, corticospinal tract, medulla and medial lemniscus.

### Initial comparison of CFA, naïve and EAE mice at long-term shows no differences in brain volume

EAE mice at day 66 had an overall smaller brain volume than both control groups. However, when these data were corrected for body weight and multiple comparisons, there was no difference in total brain volume or in any anatomical structures between EAE, Naive or CFA controls (410 ± 17 mm^3^ versus 426 ± 15 mm^3^ and 423 ± 13 mm^3^ respectively; F_(2,45)_ = 5.02 q = 0.14, ANCOVA).

### EAE mice with above average long-term clinical disease scores have decreased brain volume at long-term disease duration

In MS, atrophy is associated with disease progression, disability, and cognitive decline^9,40–42^. If this is similar in EAE mice, then it is possible that only the sickest animals have significant atrophic changes. If mice with lower chronic disease scores have little atrophy and have brain volumes similar to that of the CFA and Naïve controls, these mice could be masking any statistically significant atrophy measured in the sicker animals. We summed the clinical disease scores from day 30 onward when clinical disease scores had plateaued to obtain a cumulative long-term disease score for each mouse, which represents the total disability burden of the long-term chronic phase of the disease. We divided the EAE mice into above average “high score EAE” and below average “low score EAE” groups. Low score EAE mice had an average cumulative disease score of 103 ± 16. Average daily long-term scores were around 5.5 indicating mice exhibited tail paralysis, hind-limb weakness, and some limb dragging. High score EAE mice had a cumulative disease score of 142 ± 11. Average daily long-term scores were around 8.0 indicating mice exhibited full hind limb paralysis with some weakness in the forelimbs.

**Figure 4:**
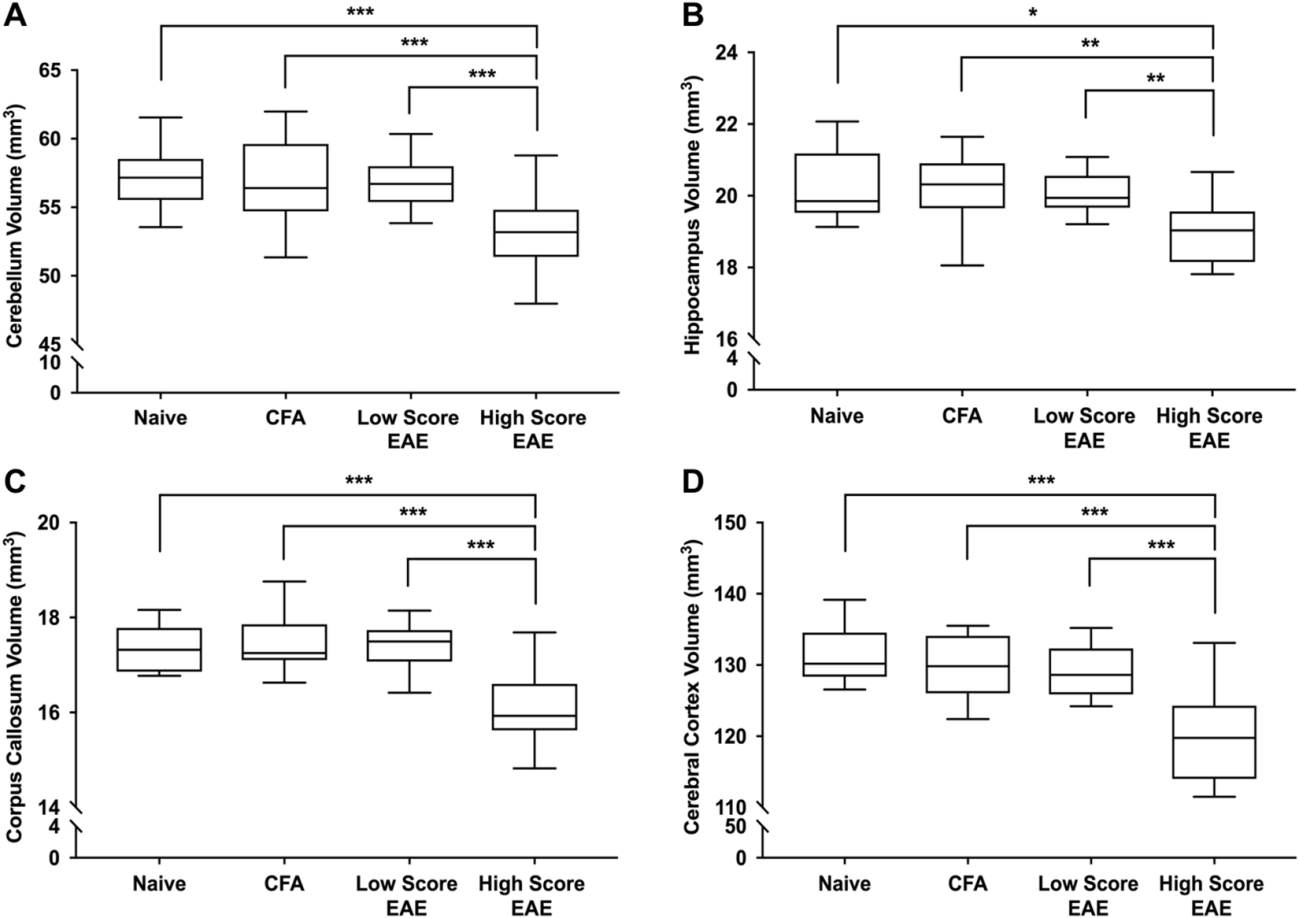
High score EAE mice have significantly smaller brains at long-term disease (day 66+). (**A**) Cerebellum volume (cerebellar cortex + arbor vita of cerebellum + cerebellar peduncle: inferior, middle, superior) in Naive, CFA, Low Score EAE and High Score EAE mice. EAE mice were divided into two groups based on cumulative long-term disease scores (summed disease scores days 30–66). Mice with below average scores were grouped as Low Score EAE and mice with above average scores were grouped as High Score EAE. (**B**) Hippocampal volume (**C**) Corpus callosal volume. (**D**) Cerebral cortex (CTX) volume (frontal lobe + parieto-temporal lobe + occipital lobe + entorhinal cortex). All volumes are adjusted based on weight using an analysis of covariance (ANCOVA). n = 9 Naïve, 13 CFA, 27 EAE, 12 Low Score EAE, 15 High Score EAE. *q < 0.05; **q < 0.01 ***q < 0.001. q values represent p values corrected for body weight and multiple comparisons via the false discovery rate (FDR) method.

After correcting for body weight, the high score EAE group had a significantly smaller total brain volume compared to the CFA, Naïve, and low score EAE groups (399 ± 16 mm^3^, 423 ± 13 mm^3^, 426 ± 15 mm^3^, and 421 ± 9 mm^3^ respectively (mean ± SD); F_(3,44)_ = 12.6, q = 0.0001, ANCOVA). We found 21 out of the 62 structures were significantly smaller in the high score EAE mice than the low score EAE mice or the CFA and Naïve controls (complete dataset in Supplementary Tables S5–8). The 21 structures were comprised of 17 GM and 4 WM structures. Structures with the biggest differences in volume were the cerebellum, hippocampus, corpus callosum, and cerebral cortex (Fig. 4). Other affected regions included the optic tract, striatum, thalamus, periaqueductal grey, basal forebrain, colliculus, and midbrain.

### Temporal decrease in brain volume in EAE mice imaged at both peak and long-term

**Figure 5:**
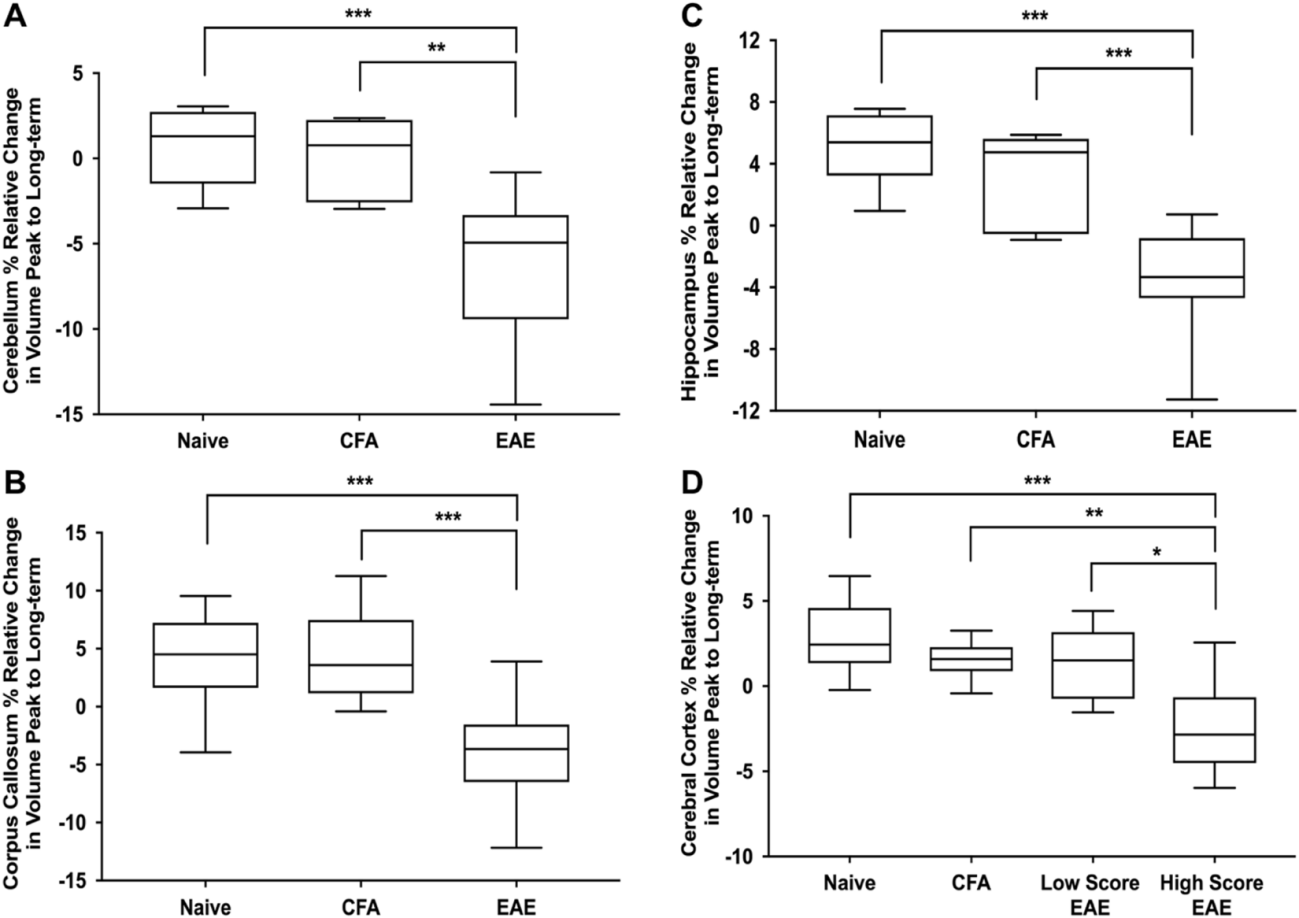
EAE mice exhibited significant reductions in brain volume from peak to long-term disease. (**A**) Relative change in cerebellum volume (cerebellar cortex + arbor vita of cerebellum + cerebellar peduncle: inferior, middle, superior) in Naive, CFA, and EAE mice. (**B**) Relative change in hippocampal volume. (**C**) Relative change in corpus callosal volume. (**D**) Relative change in cerebral cortex (CTX) volume (frontal lobe + parieto-temporal lobe + occipital lobe + entorhinal cortex) in Naive, CFA, Low Score EAE and High Score EAE mice. EAE mice were divided into two groups based on cumulative long-term disease scores (summed disease scores days 30–66). Mice with below average scores were grouped as Low Score EAE and mice with above average scores were grouped as High Score EAE. n = 9 Naïve, 7 CFA, 12 EAE, 6 Low Score EAE, 6 High Score EAE. *q < 0.05; **q < 0.01 ***q < 0.001. ANOVA test. q values represent p values corrected for multiple comparisons via the false discovery rate (FDR) method.

We next examined mice imaged at both peak and long-term clinical disease time points to measure the relative change in volume over time. Unlike comparing long-term brain volumes, we detected significant differences regarding the relative change in volume, without splitting the groups into high score and low score EAE mice. EAE mice had a significant relative decrease in total brain volume from peak to long-term compared to Naïve, and CFA mice, which increased in volume (−1.90 ± 2.69%, 4.60 ± 1.01%, 2.32 ± 1.22% respectively (mean ± SD); F_(2,22)_ = 24.8, q = 2.9E-05, ANOVA). Overall, 20 regions had significant relative decreases in volume in EAE mice (complete dataset in Supplementary Table S9). Structures with the largest differences included the cerebellum, hippocampus, and corpus callosum. When splitting the animal into high and low score groups, the only additional region that had a relative decrease in volume was the cerebral cortex in the high score mice (Fig. 5). Other affected regions included the fimbria, optic tract, basal forebrain, periaqueductal grey, colliculus, midbrain, cerebral aqueduct, and lateral ventricles.

### Correlation between the volumes of anatomical structures and behavior score

As atrophy is associated with disability in MS, we investigated whether EAE atrophy correlated with long-term (summed days 30–66) clinical disease scores.

**Figure 6:**
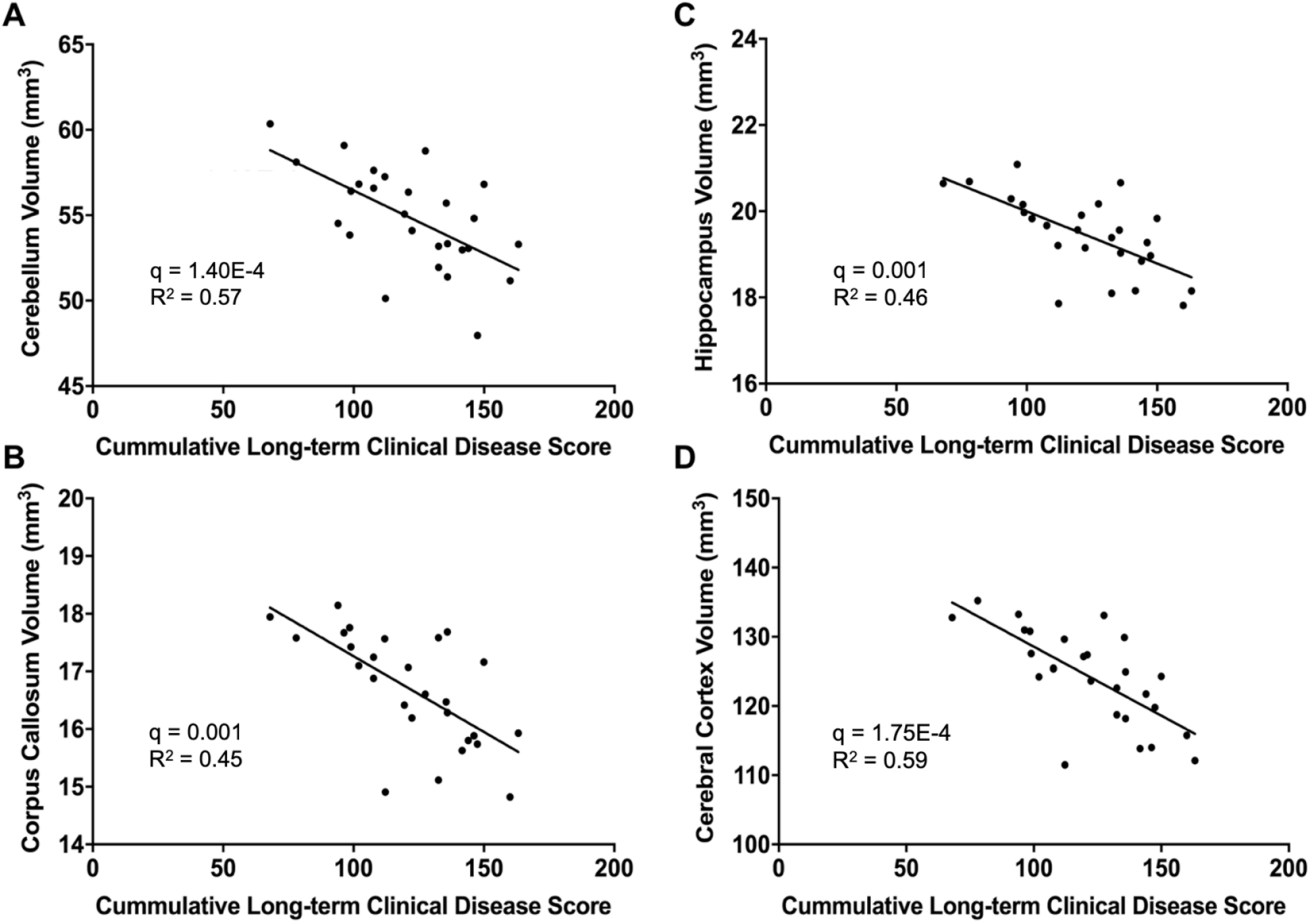
EAE mice exhibit correlations between the volumes of certain anatomical structures and long-term disease score. (**A**) Correlation of cerebellar volume (cerebellar cortex + arbor vita of cerebellum + cerebellar peduncle: inferior, middle, superior) against cumulative long-term disease score. Long-term disease scores were summed from days 30–66 to determine the total long-term burden of disease. Regressions were controlled for body weight. (**B**) Correlation of hippocampus volume. (**C**) Correlation of corpus callosum volume. (**D**) Correlation between cerebral cortex volume (frontal lobe + parieto-temporal lobe + occipital lobe + entorhinal cortex). n = 27. Linear regression test was conducted to determine significance. q values represent p values corrected for weight and multiple comparisons using the false discovery rate (FDR) method.

We performed regression analyses between absolute volume at long-term and cumulative long-term disease scores. After correcting for body weight, we identified significant correlations between long-term volume and long-term disease scores in 26 regions (Supplementary Table S10). The strongest correlations between behavior and atrophy were found in the cerebral cortex, hippocampus, cerebellum and corpus callosum (Fig. 6). Other regions included the thalamus, striatum, colliculus, periaqueductal grey, and midbrain.

**Figure 7:**
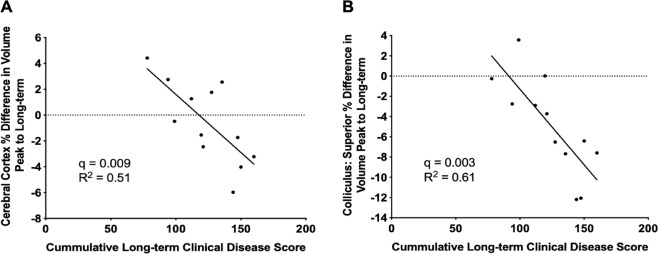
EAE mice exhibit correlations between the change in brain volume from peak to long-term and long-term disease score. (**A**) Correlation between relative change in cerebral cortex volume (frontal lobe + parieto-temporal lobe + occipital lobe + entorhinal cortex) and (**B**) hippocampus volume against cumulative long-term disease score. Long-term disease scores were summed from days 30–66 to determine the total long-term burden of disease. n = 12 for all figures. Linear regression test was conducted to determine significance. q values represent p values corrected for multiple comparisons using the false discovery rate (FDR) method.

We also undertook regression analyses between cumulative disease scores and volume loss from peak to long-term. We identified significant correlations in 14 regions (Supplementary Table S11). The strongest correlations between behavior and atrophy were found in the cerebral cortex, and the superior colliculus (Fig. 7). Other regions included the corpus callosum, hippocampus, and periaqueductal grey.

### Histological evidence of neurodegeneration in the atrophic regions: cerebral cortex, hippocampus, and cerebellum

**Figure 8:**
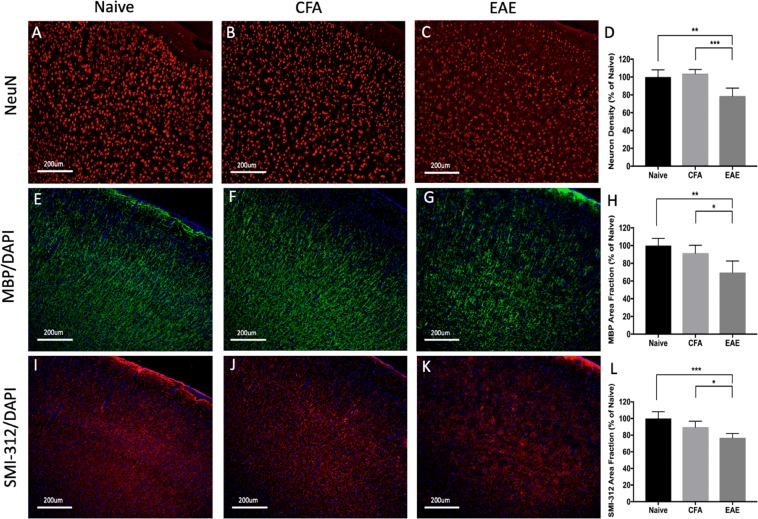
Widespread reduction of neuron, myelin, and axonal density in the cortex during experimental autoimmune encephalomyelitis (EAE) mice at long-term disease duration (66 days). Neuronal nucleus staining (NeuN - red) was significantly reduced in EAE mice (**C**) compared to Naïve (**A**) or CFA (**B**) controls. Quantification of neuron density depicted in graph (**D**). Myelin basic staining (MBP - green) was significantly reduced in EAE mice (**G**) compared to Naïve (**E**) or CFA (**F**) controls. Quantification of percent area of MBP pixel intensity depicted in graph (**H**). Axon staining (SMI-312 - red) was significantly reduced in EAE mice (**K**) compared to Naïve (**I**) or CFA (**G**) controls. Quantification of percent area of SMI-312 pixel intensity depicted in graph (**K**). DAPI – blue. n = 5 Naïve, 5 CFA, 10 EAE (NeuN), 5 EAE (SMI-312/MBP), *p < 0.05, **p < 0.01, ***p < 0.001, ANOVA test with Tukey post-hoc.

**Figure 9:**
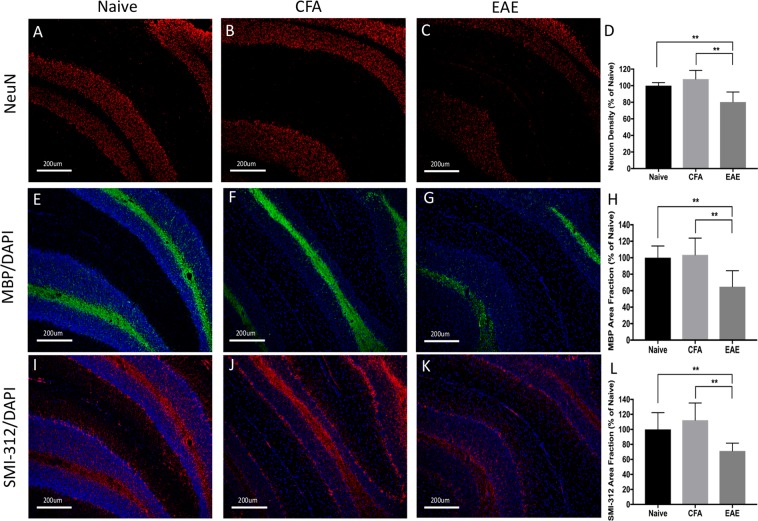
Widespread reduction of neuron, myelin, and axonal density in the cerebellum at long-term disease duration (66 days). Neuronal nucleus staining (NeuN - red) was significantly reduced in EAE mice **(C)** compared to Naïve **(A)** or CFA **(B)** controls. Quantification of neuron density depicted in graph **(D)**. Myelin basic staining (MBP - green) was significantly reduced in EAE mice **(G)** compared to Naïve **(E)** or CFA **(F)** controls. Quantification of percent area of MBP pixel intensity depicted in graph **(H)**. Axon staining (SMI-312 - red) was significantly reduced in EAE mice **(K)** compared to Naïve **(I)** or CFA **(G)** controls. Quantification of percent area of SMI-312 pixel intensity depicted in graph **(K)**. DAPI – blue. n = 5 Naïve, 5 CFA, 10 EAE (NeuN), 5 EAE (SMI-312/MBP), *p < 0.05, **p < 0.01, ***p < 0.001, ANOVA test with Tukey post-hoc.

**Figure 10:**
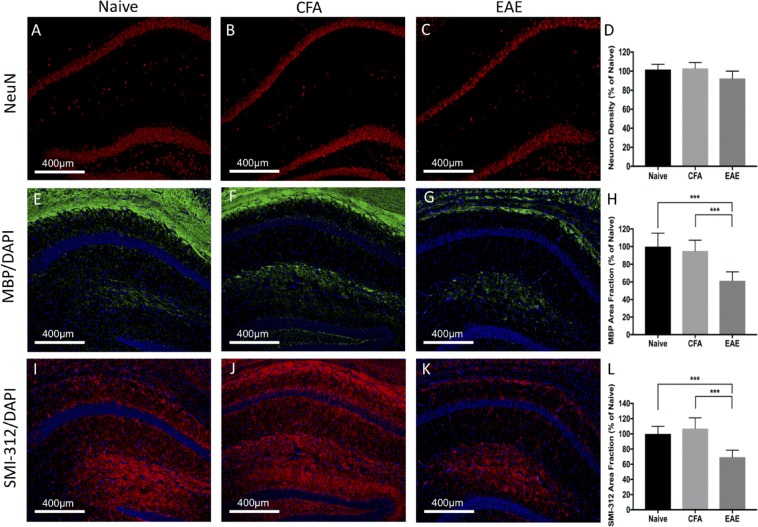
Widespread reduction of myelin, and axonal density in the hippocampus during experimental autoimmune encephalomyelitis (EAE) mice at long-term disease duration (66 days). Neuronal nucleus staining (NeuN - red) was not reduced in EAE mice (**C**) compared to Naïve (**A**) or CFA (**B**) controls. Quantification of neuron density depicted in graph (**D**). Myelin basic staining (MBP - green) was significantly reduced in EAE mice (**G**) compared to Naïve (**E**) or CFA (**F**) controls. Quantification of percent area of MBP pixel intensity depicted in graph (**H**). Axon staining (SMI-312 - red) was significantly reduced in EAE mice **(K**) compared to Naïve (**I)** or CFA **(G**) controls. Quantification of percent area of SMI-312 pixel intensity depicted in graph (**K**). DAPI – blue. n = 5 Naïve, 5 CFA, 10 EAE (NeuN), 5 EAE (SMI-312/MBP), *p < 0.05, **p < 0.01, ***p < 0.001, ANOVA test with Tukey post-hoc.

Atrophy is believed to represent the net effects of neurodegenerative processes including demyelination, axon degeneration, and neuronal death. We investigated the cortex, hippocampus, and cerebellum, which have previously reported evidence of neurodegeneration^18,21,24^, to determine whether these MRI-atrophic regions had histological evidence of neurodegenerative processes. In summary, the cortex (Fig. 8) and cerebellum (Fig. 9) had decreased density in neuronal nuclei suggesting neuronal loss though this was not seen in the hippocampus (Fig. 10). All three regions exhibited loss of myelin protein and axonal staining suggesting demyelination and axonal degeneration (Figs 8–10).

## Discussion

In MS, atrophy is believed to be the net effect of cellular degeneration and has become a clinically relevant marker of physical and cognitive decline, as well as disease progression. Inflammation, demyelination, and neurodegeneration are all interconnected in MS and likely all influence atrophy. To better understand and treat atrophy in MS, there is a need to understand the cause of atrophy and determine how it is associated with inflammation. This study is the first whole brain volumetric analysis of the EAE mouse model, an inflammatory model of demyelination commonly used to study MS. We show that the EAE mouse model exhibits localized changes in brain volume over the disease course with brain enlargement occurring at peak clinical disease followed by a reduction in volume at long-term disease.

Larger brain volumes in EAE mice at peak clinical disease suggests swelling. Increases in brain volume have also been observed during RRMS, and are believed to be due to a combination of vasogenic edema from blood brain barrier damage, gliosis, and an influx of inflammatory infiltrates from the periphery^43,44^. Regions we identified as enlarged in the EAE model, including the hippocampus, superior colliculus, midbrain and cerebellum have increased inflammatory infiltration during peak clinical disease^45–49^. As a result, it is possible that neuroinflammation may be the cause of this swelling.

Following enlargement at peak disease, EAE mice exhibit reductions in volume in multiple regions throughout the brain. This could be the result of atrophy, or for enlarged regions at peak clinical disease, could be the result of a resolution of inflammatory swelling. The latter would be similar to MS, where treatment with anti-inflammatory therapies results in volume loss referred to as psuedoatrophy^50^. To control for these options, we measured two different types of volume changes: (1) absolute brain volume comparing EAE and controls at long-term, and (2) changes in volumes over time from peak to long-term disease. A reduction in volume from peak to long-term could be dominated by resolution of swelling, while lower volumes in EAE compared to controls at long-term is predicted to be due to atrophy.

By identifying regions that showed a reduction in volume over time, and no difference at long-term between Naïve and CFA controls and EAE, one can identify regions where swelling resolved over time. These regions include the fimbria and lateral olfactory tract.

In order to conclude that atrophy has occurred we would expect that volumes would be smaller at long-term compared to controls. Twelve regions had a loss of volume over time and were also smaller compared to controls at long-term. These were likely to have had a reduction in swelling and atrophy. These include the corpus callosum, colliculus, hippocampus, optic tract, midbrain, and basal forebrain. There were 21 of 62 regions that showed a reduction in volume at long-term compared to controls indicating atrophy. These regions include the thalamus, striatum, nucleus accumbens, and frontal cortex.

There are a number of similarities between atrophy in MS, and EAE. First, there is similarities in affected regions. In MS patients, MRI studies have detected atrophy in regions including the frontal, parietal^51–53^, and visual lobes of the cerebral cortex, deep gray matter regions such as the thalamus^54^, caudate and putamen^55^, and other regions including in the hippocampus^56,57^, corpus callosum^58^, and the cerebellum^59^. We have identified evidence of atrophy in all of these structures in EAE mice.

Second, atrophy relates to progressive disability in MS^1,10,40,60^. We also saw a relationship between atrophy and motor disability in EAE. It is not surprising that our disability scores do not explain all of the variation between function and volume. This is because they are dominated by motor function tests which arguably are more sensitive to spinal damage. Other studies have found memory, and learning deficits in EAE mice at long-term disease^18,61,62^, which we now can suggest could be explained by atrophy to the hippocampus, and cerebral cortex.

Finally, atrophy in EAE is associated with neurodegeneration. We identified neurodegeneration in the cortex, hippocampus, and cerebellum. Others have found evidence of degeneration in the cortex^23,63^, hippocampus^18^, cerebellum^21^, corpus callosum^22^, superior colliculus^64^, optic tract^65^, and basal forebrain^66^. These are regions where we also found atrophy. Degeneration consisted of demyelination, as well as axonal and neuronal loss. Others have identified oligodendrocyte death as well. These affected cells/structures are similar to those that degenerate in MS^67–69^.

It is not known, how inflammation is associated with atrophy in MS. In EAE, we confirm that a central nervous system targeted autoimmune response can result in atrophy. Atrophy in EAE occurs long after the primary inflammation has subsided suggesting that the inflammation is associated with other processes which cause an accumulation of damage. Briefly, some of these possible mechanisms include demyelination^70^, activation of microglia and astrocytes^71,72^, and a loss of tissue energy^73^. Loss of axons could also be a result of Wallerian degeneration. In the case of EAE this is likely from damage to the spinal cord. A correlation between cerebral atrophy and axonal transections in the spinal cord has been reported in the EAE model^74^.

We are able to detect regional atrophy in the EAE model using a 9.4T MRI, and a Bruker cryoprobe via high resolution whole brain *in-vivo* imaging with a relatively short imaging time of 38 minutes. Atrophy has become an important measurement of disease progression in MS and an important target for future therapies. The EAE model, with this imaging protocol, has the potential to be a model for studying atrophy, and be a test bed for medications aimed at reducing the progressive deterioration of brain structures in progressive MS.

Here, we show that the EAE mouse model exhibits localized changes in brain volume over the disease course with brain enlargement occurring at peak clinical disease followed by a reduction in volume at long-term disease. From this we can draw a number of conclusions. First, that a CNS targeted autoimmune response can result in atrophy. Second, that atrophy can take place long after the peak autoimmune response has subsided and any damage to the blood brain barrier should have healed. Third, inflammation is likely associated with other processes that result in the accumulation of neurodegeneration, resulting in atrophy, as the disease progresses. These findings have implications for how inflammation may influence atrophy and disease progression in MS and for the development of future therapies.

## Supplementary information

Supplementary Tables S1-11

## Data Availability

The datasets generated during and/or analyzed during the current study are available from the corresponding author on reasonable request.
