# Supplementary material for: Central nervous system targeted autoimmunity causes regional atrophy: a 9.4T MRI study of the EAE mouse model of Multiple Sclerosis
Source: Sci Rep. 2019 Jun 11;9:8488. doi: 10.1038/s41598-019-44682-6 (PMC6560061; doi:10.1038/s41598-019-44682-6)
Supplement: Supplementary file 1 — Supplementary Tables S1-11 [file 41598_2019_44682_MOESM1_ESM.pdf]

Supplementary Data:

Central nervous system targeted autoimmunity causes regional atrophy: a 9.4T MRI study of the EAE mouse model of Multiple Sclerosis

AUTHORS: A. Max Hamilton[1,2,3], Nils D. Forkert[1,2,3], Runze Yang[1,2,3], Ying Wu[1,2,3], James A. Rogers[2,3], V. Wee Yong[2,3], and Jeff F. Dunn [1,2,3]

INSTITUTIONS:

[1]Department of Radiology, [2]Hotchkiss Brain Institute, [3]Department of Clinical Neurosciences,
Cumming School of Medicine
University of Calgary
Calgary, Alberta, Canada

Supplementary Table S1. Uncorrected volumes of brain structures at peak clinical EAE disease.

Anatomical Region	Volume, mean ± SD mm^3		
	Naïve	CFA	EAE
**White Matter**			
Fimbria	2.93 ± 0.14	2.98 ± 0.14	3.26 ± 0.17
Corpus Callosum	16.4 ± 0.6	16.6 ± 0.6	17.4 ± 0.5
Posterior Commissure	0.11 ± 0.01	0.11 ± 0.01	0.13 ± 0.01
Fornix	0.60 ± 0.05	0.59 ± 0.04	0.65 ± 0.05
Stria Medullaris	0.66 ± 0.05	0.65 ± 0.04	0.69 ± 0.03
Optic Tract	1.40 ± 0.07	1.38 ± 0.06	1.46 ± 0.08
Fasciculus Retroflexus	0.24 ± 0.01	0.23 ± 0.01	0.25 ± 0.01
Anterior Commissure: Pars Anterior	1.22 ± 0.04	1.19 ± 0.05	1.24 ± 0.06
Internal Capsule	2.31 ± 0.15	2.22 ± 0.15	2.37 ± 0.15
Anterior Commissure: Pars Posterior	0.38 ± 0.04	0.36 ± 0.03	0.38 ± 0.04
Stria Terminalis	0.81 ± 0.05	0.77 ± 0.04	0.79 ± 0.04
Habenular Commissure	0.03 ± 0.01	0.03 ± 0.01	0.03 ± 0.01
Mammilothalamic Tract	0.22 ± 0.01	0.22 ± 0.02	0.22 ± 0.02
**Grey Matter**			
Lateral Septum	2.78 ± 0.11	2.77 ± 0.12	3.04 ± 0.14
Basal Forebrain	3.93 ± 0.19	3.83 ± 0.21	4.21 ± 0.20
Periaqueductal Grey	3.58 ± 0.16	3.57 ± 0.26	3.87 ± 0.11
Hippocampus	18.9 ± 1.1	19.1 ± 1.2	20.4 ± 0.6
Cerebral Peduncle	1.88 ± 0.09	1.87 ± 0.10	2.03 ± 0.15
Amygdala	11.4 ± 0.6	11.4 ± 0.8	12.0 ± 0.5
Dentate Gyrus of Hippocampus	3.32 ± 0.21	3.39 ± 0.19	3.54 ± 0.13
Medial Septum	0.92 ± 0.07	0.86 ± 0.06	0.93 ± 0.07
Stratum Granulosum of Hippocampus	0.80 ± 0.05	0.84 ± 0.05	0.86 ± 0.02
Nucleus Accumbens	3.28 ± 0.19	3.21 ± 0.16	3.38 ± 0.18
Hypothalamus	9.19 ± 0.49	9.00 ± 0.34	9.52 ± 0.79
Fundus of Striatum	0.11 ± 0.02	0.11 ± 0.02	0.12 ± 0.02
Thalamus	15.6 ± 0.7	15.4 ± 0.6	15.8 ± 0.4
Pre-Para Subiculum	2.15 ± 0.12	2.12 ± 0.13	2.18 ± 0.07
Globus Pallidus	2.40 ± 0.19	2.30 ± 0.12	2.38 ± 0.14
Bed Nucleus of Stria Terminalis	1.19 ± 0.07	1.16 ± 0.07	1.20 ± 0.08
Striatum	17.7 ± 0.9	17.7 ± 0.8	18.1 ± 0.8
Mammillary Bodies	0.46 ± 0.04	0.45 ± 0.03	0.47 ± 0.04

Supplementary Table S1. (Continued) Uncorrected volumes of brain structures at peak clinical EAE disease.

Anatomical Region	Volume, mean ± SD mm^3		
	Naïve	CFA	EAE
**Ventricles**			
Cerebral Aqueduct	0.43 ± 0.04	0.48 ± 0.04	0.52 ± 0.03
Lateral Ventricle	3.32 ± 0.18	3.44 ± 0.17	3.85 ± 0.29
Third Ventricle	0.98 ± 0.06	1.02 ± 0.06	1.11 ± 0.08
Fourth Ventricle	0.47 ± 0.04	0.40 ± 0.04	0.51 ± 0.06
**Cerebral Cortex**			
Entorhinal Cortex	10.3 ± 0.5	10.6 ± 0.4	10.8 ± 0.2
Parieto-Temporal Lobe	71.1 ± 2.8	71.0 ± 3.6	72.6 ± 2.0
Occipital Lobe	5.98 ± 0.33	5.85 ± 0.36	5.92 ± 0.18
Frontal Lobe	39.0 ± 1.3	39.2 ± 1.6	39.2 ± 0.8
**Brainstem**			
Pons	14.1 ± 0.9	13.8 ± 0.5	15.0 ± 0.6
Superior Olivary Complex	0.55 ± 0.08	0.56 ± 0.05	0.62 ± 0.03
Corticospinal Tract/Pyramids	1.11 ± 0.07	1.18 ± 0.10	1.27 ± 0.10
Colliculus: Superior	7.72 ± 0.35	7.63 ± 0.38	8.01 ± 0.19
Pontine Nucleus	0.59 ± 0.05	0.61 ± 0.04	0.66 ± 0.05
Medial Lemniscus/Medial Longitudinal Fasciculus	2.04 ± 0.15	2.04 ± 0.12	2.19 ± 0.13
Cuneate Nucleus	0.24 ± 0.03	0.25 ± 0.02	0.28 ± 0.03
Ventral Tegmental Decussation	0.11 ± 0.01	0.09 ± 0.01	0.11 ± 0.01
Medulla	21.9 ± 1.7	21.7 ± 1.1	23.7 ± 1.8
Midbrain	11.4 ± 0.82	11.4 ± 0.40	12.0 ± 0.23
Inferior Olivary Complex	0.25 ± 0.03	0.27 ± 0.03	0.29 ± 0.02
Colliculus: Inferior	5.21 ± 0.25	5.21 ± 0.23	5.41 ± 0.16
Interpeduncular Nucleus	0.23 ± 0.03	0.23 ± 0.02	0.25 ± 0.03
Facial Nerve (Cranial Nerve 7)	0.21 ± 0.02	0.20 ± 0.01	0.21 ± 0.03
**Cerebellum**			
Arbor Vita of Cerebellum	8.71 ± 0.62	8.50 ± 0.40	9.24 ± 0.31
Cerebellar Peduncle: Inferior	0.66 ± 0.06	0.68 ± 0.05	0.75 ± 0.06
Cerebellar Peduncle: Middle	0.98 ± 0.06	0.99 ± 0.05	1.06 ± 0.06
Cerebellar Cortex	44.7 ± 2.6	45.2 ± 2.1	47.0 ± 1.1
Cerebellar Peduncle: Superior	0.92 ± 0.04	0.86 ± 0.04	0.92 ± 0.03
**Olfactory**			
Lateral Olfactory Tract	1.15 ± 0.05	1.14 ± 0.04	1.21 ± 0.03
Olfactory Tubercle	3.19 ± 0.23	3.04 ± 0.17	3.33 ± 0.12
Olfactory Bulbs	21.1 ± 1.1	21.3 ± 1.0	21.5 ± 01.1
Subependymale Zone / Rhinocele	0.06 ± 0.01	0.06 ± 0.01	0.06 ± 0.01

Supplementary Table S1. (Continued) Uncorrected volumes of brain structures at peak clinical EAE disease.

Cumulative Anatomical Region	Volume, mean ± SD mm^3		
	Naïve	CFA	EAE
Ventricles	5.19 ± 0.28	5.33 ± 0.23	6.00 ± 0.37
Cerebral Cortex	29.2 ± 1.2	29.2 ± 1.3	30.9 ± 0.9
Cerebral White	69.2 ± 4.4	68.8 ± 2.8	73.8 ± 1.4
Cerebellum	56.0 ± 3.4	56.2 ± 2.5	58.9 ± 1.4
Total Brain Volume	405 ± 18	405 ± 15	422 ± 9
Brain Stem	220 ± 9	220 ± 9	227 ± 5
Cerebral Grey	25.5 ± 1.1	25.6 ± 1.1	26.1 ± 1.2
Olfactory	126 ± 5	127 ± 5	129 ± 2

Volumes mean ± standard deviation.

Supplementary Table S2. Volumes of brain structures corrected for body weight, at peak clinical EAE disease.

Anatomical Region	Volume, mean ± SD mm^3		
	Naïve	CFA	EAE
**White Matter**			
Fimbria	2.96 ± 0.14	2.98 ± 0.14	3.25 ± 0.17
Corpus Callosum	16.5 ± 0.6	16.5 ± 0.6	17.4 ± 0.5
Posterior Commissure	0.11 ± 0.01	0.11 ± 0.01	0.13 ± 0.01
Fornix	0.60 ± 0.05	0.59 ± 0.04	0.65 ± 0.05
Stria Medullaris	0.67 ± 0.05	0.65 ± 0.04	0.69 ± 0.03
Optic Tract	1.40 ± 0.07	1.38 ± 0.06	1.46 ± 0.08
Fasciculus Retroflexus	0.24 ± 0.01	0.23 ± 0.01	0.25 ± 0.01
Anterior Commissure: Pars Anterior	1.22 ± 0.04	1.19 ± 0.05	1.24 ± 0.06
Internal Capsule	2.31 ± 0.15	2.22 ± 0.15	2.37 ± 0.15
Anterior Commissure: Pars Posterior	0.38 ± 0.04	0.36 ± 0.03	0.38 ± 0.04
Stria Terminalis	0.81 ± 0.05	0.77 ± 0.04	0.79 ± 0.04
Habenular Commissure	0.03 ± 0.01	0.03 ± 0.01	0.03 ± 0.01
Mammilothalamic Tract	0.22 ± 0.01	0.22 ± 0.02	0.23 ± 0.02
**Grey Matter**			
Lateral Septum	2.80 ± 0.11	2.77 ± 0.12	3.04 ± 0.14
Basal Forebrain	3.92 ± 0.19	3.83 ± 0.21	4.21 ± 0.20
Periaqueductal Grey	3.61 ± 0.16	3.56 ± 0.26	3.86 ± 0.11
Hippocampus	19.0 ± 1.1	19.1 ± 1.2	20.3 ± 0.6
Cerebral Peduncle	1.88 ± 0.09	1.87 ± 0.10	2.03 ± 0.15
Amygdala	11.4 ± 0.6	11.4 ± 0.8	12.0 ± 0.5
Dentate Gyrus of Hippocampus	3.34 ± 0.21	3.38 ± 0.19	3.53 ± 0.13
Medial Septum	0.92 ± 0.07	0.86 ± 0.06	0.93 ± 0.07
Stratum Granulosum of Hippocampus	0.81 ± 0.05	0.83 ± 0.05	0.86 ± 0.02
Nucleus Accumbens	3.26 ± 0.19	3.22 ± 0.16	3.38 ± 0.18
Hypothalamus	9.15 ± 0.49	9.01 ± 0.34	9.53 ± 0.79
Fundus of Striatum	0.12 ± 0.02	0.11 ± 0.02	0.12 ± 0.02
Thalamus	15.6 ± 0.7	15.4 ± 0.6	15.8 ± 0.4
Pre-Para Subiculum	2.16 ± 0.12	2.12 ± 0.13	2.17 ± 0.07
Globus Pallidus	2.39 ± 0.19	2.31 ± 0.12	2.38 ± 0.14
Bed Nucleus of Stria Terminalis	1.19 ± 0.07	1.16 ± 0.07	1.21 ± 0.08
Striatum	17.7 ± 0.9	17.7 ± 0.8	18.1 ± 0.8
Mammillary Bodies	0.46 ± 0.04	0.45 ± 0.03	0.47 ± 0.04

Supplementary Table S2. (Continued) Volumes of brain structures corrected for body weight, at peak clinical EAE disease.

Anatomical Region	Volume, mean ± SD mm^3		
	Naïve	CFA	EAE
**Ventricles**			
Lateral Ventricle	3.35 ± 0.18	3.43 ± 0.17	3.84 ± 0.29
Cerebral Aqueduct	0.43 ± 0.04	0.47 ± 0.04	0.52 ± 0.03
Third Ventricle	0.98 ± 0.06	1.02 ± 0.06	1.11 ± 0.08
Fourth Ventricle	0.47 ± 0.04	0.40 ± 0.04	0.51 ± 0.06
**Cerebral Cortex**			
Entorhinal Cortex	10.4 ± 0.5	10.6 ± 0.4	10.8 ± 0.2
Parieto-Temporal Lobe	71.5 ± 2.8	71.0 ± 3.6	72.4 ± 2.0
Occipital Lobe	6.02 ± 0.33	5.84 ± 0.36	5.90 ± 0.18
Frontal Lobe	39.1 ± 1.3	39.1 ± 1.6	39.2 ± 0.8
**Brainstem**			
Pons	14.1 ± 0.9	13.8 ± 0.5	15 ± 0.6
Superior Olivary Complex	0.55 ± 0.08	0.56 ± 0.05	0.62 ± 0.03
Corticospinal Tract/Pyramids	1.11 ± 0.07	1.18 ± 0.10	1.28 ± 0.10
Medulla	21.8 ± 1.7	21.7 ± 1.1	23.7 ± 1.8
Pontine Nucleus	0.59 ± 0.05	0.61 ± 0.04	0.66 ± 0.05
Medial Lemniscus/Medial Longitudinal Fasciculus	2.04 ± 0.15	2.04 ± 0.12	2.19 ± 0.13
Midbrain	11.5 ± 0.8	11.4 ± 0.4	12 ± 0.2
Colliculus: Superior	7.77 ± 0.35	7.62 ± 0.38	7.99 ± 0.19
Ventral Tegmental Decussation	0.11 ± 0.01	0.09 ± 0.01	0.11 ± 0.01
Inferior Olivary Complex	0.25 ± 0.03	0.27 ± 0.03	0.29 ± 0.02
Cuneate Nucleus	0.26 ± 0.03	0.25 ± 0.02	0.28 ± 0.03
Colliculus: Inferior	5.23 ± 0.25	5.20 ± 0.23	5.40 ± 0.16
Interpeduncular Nucleus	0.23 ± 0.03	0.23 ± 0.02	0.25 ± 0.03
Facial Nerve (Cranial Nerve 7)	0.21 ± 0.02	0.20 ± 0.01	0.21 ± 0.03
**Cerebellum**			
Arbor Vita of Cerebellum	8.76 ± 0.62	8.49 ± 0.40	9.22 ± 0.31
Cerebellar Peduncle: Inferior	0.66 ± 0.06	0.68 ± 0.05	0.75 ± 0.06
Cerebellar Peduncle: Middle	0.98 ± 0.06	0.99 ± 0.05	1.06 ± 0.06
Cerebellar Cortex	45.0 ± 2.6	45.1 ± 2.1	46.9 ± 1.1
Cerebellar Peduncle: Superior	0.93 ± 0.04	0.86 ± 0.04	0.92 ± 0.03
**Olfactory**			
Lateral Olfactory Tract	1.15 ± 0.05	1.14 ± 0.04	1.21 ± 0.03
Olfactory Tubercle	3.20 ± 0.23	3.04 ± 0.17	3.33 ± 0.12
Olfactory Bulbs	21.0 ± 1.1	21.3 ± 1.0	21.5 ± 1.1
Subependymale Zone / Rhinocele	0.06 ± 0.01	0.06 ± 0.01	0.06 ± 0.01

Supplementary Table S2. (Continued) Volumes of brain structures corrected for body weight, at peak clinical EAE disease.

Cumulative Anatomical Region	Volume, mean ± SD mm^3		
	Naïve	CFA	EAE
Ventricles	5.22 ± 0.28	5.32 ± 0.23	5.98 ± 0.37
Cerebral White	29.3 ± 1.2	29.1 ± 1.3	30.9± 0.9
Brain Stem	69.3 ± 4.4	68.7 ± 2.8	73.7 ± 1.4
Cerebellum Total	56.3 ± 3.4	56.2 ± 2.5	58.8 ± 1.4
Total Brain Volume	407 ± 18	405 ± 15	422 ± 9
Cerebral Grey	221 ± 9	220 ± 9	226 ± 5
Olfactory	25.4 ± 1.1	25.6 ± 1.1	26.1 ± 1.2
Cerebral Cortex	127 ± 5	127 ± 5	128 ± 3

Volumes mean ± standard deviation corrected for body weight using an ANCOVA analysis.

Supplementary Table S3. Comparing the uncorrected volumes of Naïve, CFA, and EAE mice at peak clinical disease.

Anatomical Region	% Difference in Between Groups					
	Naïve vs. CFA	*q-value*	Naïve vs. EAE	*q-value*	CFA vs. EAE	*q-value*
**White Matter**						
Fimbria	-1.6	0.680	9.3	0.008	11.1	0.009
Corpus Callosum	-1.0	0.779	5.1	0.031	6.1	0.010
Cerebral Peduncle	0.6	0.884	8.6	0.027	7.9	0.078
Posterior Commissure	-2.3	0.779	14.4	0.036	17.1	0.039
Fornix	1.7	0.761	9.2	0.029	7.4	0.128
Stria Medullaris	2.4	0.667	7.0	0.025	4.4	0.220
Optic Tract	1.3	0.763	5.6	0.044	4.3	0.193
Fasciculus Retroflexus	5.0	0.208	6.6	0.076	1.6	0.750
Anterior Commissure: Pars Anterior	2.7	0.259	4.1	0.088	1.3	0.721
Internal Capsule	4.0	0.342	6.5	0.079	2.3	0.673
Anterior Commissure: Pars Posterior	7.1	0.268	6.9	0.185	-0.2	0.994
Stria Terminalis	4.9	0.207	3.1	0.265	-1.7	0.729
Habenular Commissure	-7.8	0.440	2.6	0.861	11.3	0.375
Mammilothalamic Tract	-1.0	0.876	1.9	0.761	2.9	0.685
**Grey Matter**						
Lateral Septum	0.5	0.888	9.9	0.003	9.3	0.011
Basal Forebrain	2.6	0.401	10.1	0.005	7.3	0.036
Periaqueductal Grey	0.4	0.949	8.6	0.012	8.1	0.006
Hippocampus	-1.3	0.779	6.6	0.024	8.0	0.008
Amygdala	-0.1	0.995	5.8	0.055	5.9	0.055
Dentate Gyrus of Hippocampus	-1.8	0.686	4.5	0.081	6.4	0.040
Medial Septum	7.2	0.159	8.1	0.055	0.9	0.891
Stratum Granulosum of Hippocampus	-3.7	0.310	3.1	0.292	7.1	0.054
Nucleus Accumbens	1.9	0.679	5.0	0.080	3.1	0.357
Hypothalamus	2.0	0.425	5.7	0.117	3.6	0.405
Fundus of Striatum	8.1	0.407	13.3	0.194	4.9	0.763
Thalamus	0.9	0.780	2.4	0.137	1.5	0.426
Pre-Para Subiculum	1.3	0.783	2.8	0.267	1.5	0.473
Globus Pallidus	4.1	0.316	3.2	0.269	-0.9	0.885
Bed Nucleus of Stria Terminalis	2.9	0.410	3.7	0.265	0.8	0.887
Striatum	-0.3	0.950	2.1	0.352	2.4	0.364
Mammillary Bodies	1.2	0.872	3.6	0.396	2.4	0.762

Supplementary Table S3. (Continued) Comparing the uncorrected volumes of Naïve, CFA, and EAE mice at peak clinical disease.

	% Difference in Between Groups					
Anatomical Region	Naïve vs. CFA	*q-value*	Naïve vs. EAE	*q-value*	CFA vs. EAE	*q-value*
**Ventricles**						
Cerebral Aqueduct	-10.5	0.053	9.0	0.043	21.9	0.006
Lateral Ventricle	-3.4	0.270	12.0	0.008	15.9	0.008
Third Ventricle	-4.3	0.216	9.9	0.017	14.8	0.010
Fourth Ventricle	17.9	0.023	27.5	0.004	8.1	0.250
**Cerebral Cortex**						
Entorhinal Cortex	-2.9	0.270	1.5	0.352	4.6	0.043
Parieto-Temporal Lobe	0.0	0.999	2.2	0.296	2.2	0.259
Occipital Lobe	2.3	0.474	1.1	0.742	-1.1	0.745
Frontal Lobe	-0.4	0.891	0.1	0.954	0.6	0.781
**Brain Stem**						
Pons	1.7	0.690	8.1	0.004	6.3	0.076
Superior Olivary Complex	-2.2	0.779	11.0	0.011	13.5	0.030
Corticospinal Tract/Pyramids	-5.6	0.227	8.1	0.078	14.5	0.013
Colliculus: Superior	1.1	0.781	5.0	0.026	3.9	0.067
Pontine Nucleus	-4.2	0.341	7.3	0.078	12.0	0.037
Medial Lemniscus/Medial Longitudinal Fasciculus	0.1	0.996	7.5	0.034	7.4	0.079
Cuneate Nucleus	-3.3	0.476	10.1	0.081	13.9	0.075
Ventral Tegmental Decussation	20.0	0.044	14.1	0.044	-4.9	0.353
Medulla	0.8	0.886	9.1	0.027	8.3	0.109
Midbrain	0.3	0.946	5.2	0.010	4.9	0.101
Inferior Olivary Complex	-7.8	0.312	7.4	0.181	16.4	0.024
Colliculus: Inferior	0.0	1.000	3.8	0.069	3.8	0.110
Interpeduncular Nucleus	-0.7	0.949	6.1	0.213	6.8	0.320
Facial Nerve (Cranial Nerve 7)	3.2	0.502	5.1	0.353	1.9	0.870
**Cerebellum**						
Arbor Vita of Cerebellum	2.5	0.397	8.6	0.004	6.0	0.036
Cerebellar Peduncle: Inferior	-3.2	0.423	10.7	0.024	14.4	0.013
Cerebellar Peduncle: Middle	-0.5	0.914	7.6	0.025	8.1	0.054
Cerebellar Cortex	-1.1	0.778	3.9	0.055	5.0	0.036
Cerebellar Peduncle: Superior	7.4	0.036	6.8	0.011	-0.6	0.872
**Olfactory**						
Lateral Olfactory Tract	0.8	0.780	6.0	0.005	5.2	0.021
Olfactory Tubercle	4.8	0.236	9.7	0.004	4.7	0.159
Olfactory Bulbs	-1.0	0.780	0.8	0.800	1.9	0.473
Subependymale Zone / Rhinocele	3.2	0.774	4.2	0.456	0.9	0.948

Supplementary Table S3. (Continued) Comparing the uncorrected volumes of Naïve, CFA, and EAE mice at peak clinical disease.

Anatomical Region	% Difference in Between Groups					
	Naïve vs. CFA	*q-value*	Naïve vs. EAE	*q-value*	CFA vs. EAE	*q-value*
Ventricles	-2.6	0.342	12.5	0.004	15.5	0.004
Cerebral White	0.1	0.992	5.9	0.012	5.8	0.024
Brain Stem	0.6	0.884	7.3	0.006	6.6	0.043
Cerebellum Total	-0.4	0.917	4.8	0.025	5.3	0.033
Total Brain Volume	3.7E-03	0.998	4.2	0.024	4.2	0.044
Cerebral Grey	-7.0E-03	0.999	2.8	0.096	2.8	0.111
Olfactory	-0.2	0.954	2.1	0.343	2.4	0.356
Cerebral Cortex	-0.3	0.949	1.4	0.376	1.7	0.271

% volume difference compares average volume between groups (positive indicates increase in size, negative indicates decrease in size). q-values (significance = <0.1) represent FDR-adjusted p-values.

Supplementary Table S4. Comparing volumes, corrected for body weight, of Naïve, CFA, and EAE mice at peak clinical disease.

Anatomical Region	% Difference in Between Groups					
	Naïve vs. CFA	*q-value*	Naïve vs. EAE	*q-value*	CFA vs. EAE	*q-value*
**White Matter**						
Fimbria	0.5	0.882	9.7	0.002	9.2	0.003
Corpus Callosum	0.5	0.871	5.5	0.024	5.0	0.031
Cerebral Peduncle	-0.4	0.936	8.2	0.024	8.6	0.048
Posterior Commissure	1.2	0.896	15.7	0.029	14.3	0.038
Fornix	-1.5	0.798	7.6	0.030	9.3	0.093
Stria Medullaris	-2.9	0.388	3.8	0.038	6.9	0.275
Optic Tract	-1.3	0.756	4.2	0.048	5.6	0.188
Fasciculus Retroflexus	-5.1	0.192	1.1	0.062	6.6	0.815
Anterior Commissure: Pars Anterior	-2.6	0.319	1.4	0.081	4.1	0.696
Internal Capsule	-3.9	0.343	2.3	0.086	6.5	0.676
Anterior Commissure: Pars Posterior	-7.0	0.249	-0.7	0.215	6.8	0.920
Stria Terminalis	-4.7	0.193	-1.7	0.306	3.1	0.723
Habenular Commissure	8.0	0.488	10.7	0.854	2.6	0.389
Mammilothalamic Tract	-3.6	0.798	0.3	0.730	4.1	0.417
**Grey Matter**						
Basal Forebrain	-2.4	0.418	7.4	0.003	10.1	0.035
Lateral Septum	-1.1	0.780	8.7	0.002	9.8	0.008
Periaqueductal Grey	-1.3	0.737	7.0	0.004	8.5	0.029
Hippocampus	0.4	0.900	6.9	0.012	6.5	0.021
Amygdala	-0.8	0.843	4.8	0.041	5.7	0.118
Dentate Gyrus of Hippocampus	1.2	0.760	5.6	0.080	4.4	0.063
Medial Septum	-6.1	0.194	1.6	0.059	8.2	0.791
Stratum Granulosum of Hippocampus	3.1	0.356	6.3	0.283	3.0	0.080
Nucleus Accumbens	-1.5	0.752	3.5	0.084	5.1	0.287
Hypothalamus	-1.5	0.779	4.2	0.098	5.8	0.289
Fundus of Striatum	-8.3	0.413	3.8	0.194	13.2	0.792
Thalamus	-1.1	0.717	1.3	0.195	2.4	0.486
Pre-Para Subiculum	-1.9	0.460	0.8	0.262	2.7	0.820
Globus Pallidus	-3.5	0.345	-0.3	0.314	3.3	0.939
Bed Nucleus of Stria Terminalis	-2.4	0.487	1.3	0.263	3.8	0.794
Striatum	0.2	0.938	2.3	0.365	2.1	0.389
Mammillary Bodies	-0.9	0.895	2.7	0.394	3.6	0.721

Supplementary Table S4. (Continued) Comparing volumes, corrected for body weight, of Naïve, CFA, and EAE mice at peak clinical disease.

Anatomical Region	% Difference in Between Groups					
	Naïve vs. CFA	*q-value*	Naïve vs. EAE	*q-value*	CFA vs. EAE	*q-value*
**Ventricles**						
Lateral Ventricle	2.5	0.467	14.6	0.003	11.9	0.002
Cerebral Aqueduct	10.5	0.048	20.4	0.033	8.9	0.003
Third Ventricle	4.1	0.328	14.3	0.014	9.8	0.005
Fourth Ventricle	-14.2	0.042	9.4	0.002	27.6	0.152
**Cerebral Cortex**						
Entorhinal Cortex	2.5	0.247	4.0	0.388	1.4	0.073
Parieto-Temporal Lobe	-0.7	0.789	1.3	0.250	2.1	0.475
Occipital Lobe	-3.0	0.269	-2.0	0.749	1.0	0.419
Frontal Lobe	1.7	0.924	3.7	0.950	2.0	0.897
**Brain Stem**						
Pons	-1.8	0.655	6.1	0.009	8.1	0.055
Superior Olivary Complex	2.6	0.730	14.0	0.029	11.0	0.025
Corticospinal Tract/Pyramids	6.0	0.250	14.6	0.060	8.1	0.014
Medulla	-0.7	0.897	8.4	0.030	9.2	0.072
Pontine Nucleus	4.4	0.364	12.0	0.081	7.3	0.030
Medial Lemniscus/Medial Longitudinal Fasciculus	-0.1	0.981	7.4	0.038	7.5	0.070
Midbrain	-0.7	0.845	4.5	0.033	5.2	0.092
Colliculus: Superior	-2.0	0.309	2.9	0.012	4.9	0.149
Ventral Tegmental Decussation	-16.8	0.026	-5.0	0.047	14.1	0.406
Inferior Olivary Complex	8.1	0.267	16.0	0.198	7.3	0.039
Cuneate Nucleus	-2.0	0.755	8.0	0.078	10.2	0.059
Colliculus: Inferior	-0.5	0.873	3.2	0.073	3.7	0.188
Interpeduncular Nucleus	0.6	0.940	6.7	0.275	6.1	0.306
Facial Nerve (Cranial Nerve 7)	-3.0	0.752	2.0	0.366	5.2	0.814
**Cerebellum**						
Arbor Vita of Cerebellum	-3.0	0.246	5.3	0.002	8.6	0.048
Cerebellar Peduncle: Inferior	3.2	0.468	14.2	0.021	10.7	0.013
Cerebellar Peduncle: Middle	0.6	0.896	8.2	0.030	7.6	0.041
Cerebellar Cortex	0.3	0.899	4.1	0.043	3.8	0.058
Cerebellar Peduncle: Superior	-7.8	0.003	-1.6	0.004	6.6	0.388
**Olfactory**						
Lateral Olfactory Tract	-0.8	0.758	5.1	0.004	6.0	0.025
Olfactory Tubercle	-5.0	0.136	4.2	0.005	9.6	0.198
Olfactory Bulbs	1.5	0.724	2.4	0.784	0.9	0.388
Subependymale Zone / Rhinocele	-3.6	0.798	0.3	0.730	4.1	0.417

Supplementary Table S4. (Continued) Comparing volumes, corrected for body weight, of Naïve, CFA, and EAE mice at peak clinical disease.

Anatomical Region	% Difference in Between Groups					
	Naïve vs. CFA	*q-value*	Naïve vs. EAE	*q-value*	CFA vs. EAE	*q-value*
Ventricles	-0.3	0.671	1.1	0.002	1.4	0.003
Cerebral White	-0.3	0.870	4.4	0.034	4.7	0.011
Brain Stem	-0.4	0.815	3.7	0.038	4.1	0.011
Cerebellum Total	-0.4	0.901	2.3	0.046	2.8	0.019
Total Brain Volume	-0.5	0.875	5.3	0.072	5.9	0.029
Cerebral Grey	1.9	0.869	14.6	0.235	12.4	0.098
Olfactory	0.5	0.875	2.7	0.302	2.2	0.322
Cerebral Cortex	-0.9	0.898	6.3	0.662	7.3	0.363

% volume difference compares average volume between groups (positive indicates increase in size, negative indicates decrease in size). q-values (significance = <0.1) represent FDR-adjusted p-values.

Supplementary Table S5. Uncorrected volumes of brain structures at long-term EAE disease duration.

Anatomical Regions	Volume, mean ± SD mm^3			
	Naïve	CFA	Low Score EAE	High Score EAE
**White Matter**				
Corpus Callosum	17.4 ± 0.5	17.5 ± 0.6	17.4 ± 0.5	16.1 ± 0.9
Stria Terminalis	0.82 ± 0.05	0.85 ± 0.03	0.83 ± 0.06	0.77 ± 0.05
Optic Tract	1.48 ± 0.04	1.51 ± 0.06	1.46 ± 0.06	1.40 ± 0.07
Anterior Commissure: Pars Anterior	1.28 ± 0.06	1.28 ± 0.06	1.27 ± 0.08	1.21 ± 0.05
Internal Capsule	2.43 ± 0.12	2.45 ± 0.11	2.38 ± 0.20	2.28 ± 0.15
Stria Medullaris	0.72 ± 0.04	0.70 ± 0.04	0.71 ± 0.05	0.67 ± 0.04
Fasciculus Retroflexus	0.24 ± 0.02	0.25 ± 0.02	0.25 ± 0.01	0.23 ± 0.02
Fimbria	3.13 ± 0.18	3.18 ± 0.16	3.22 ± 0.10	3.05 ± 0.21
Anterior Commissure: Pars Posterior	0.41 ± 0.04	0.40 ± 0.03	0.39 ± 0.04	0.37 ± 0.04
Fornix	0.63 ± 0.03	0.63 ± 0.02	0.62 ± 0.04	0.61 ± 0.03
Habenular Commissure	0.03 ± 0.01	0.03 ± 0.01	0.03 ± 0.01	0.03 ± 0.01
Posterior Commissure	0.12 ± 0.01	0.12 ± 0.01	0.13 ± 0.01	0.12 ± 0.02
Mammilothalamic Tract	0.23 ± 0.01	0.23 ± 0.01	0.23 ± 0.01	0.23 ± 0.01
Cerebral Peduncle	2.03 ± 0.09	2.02 ± 0.09	2.03 ± 0.07	2.00 ± 0.14
**Grey Matter**				
Striatum	18.5 ± 0.7	18.4 ± 0.6	18.4 ± 0.6	17.2 ± 0.8
Thalamus	16.1 ± 0.7	16.3 ± 0.3	16.3 ± 0.5	15.4 ± 0.5
Pre-Para Subiculum	2.26 ± 0.11	2.23 ± 0.10	2.20 ± 0.09	2.07 ± 0.11
Periaqueductal Grey	3.68 ± 0.25	3.82 ± 0.16	3.74 ± 0.09	3.48 ± 0.24
Nucleus Accumbens	3.47 ± 0.17	3.44 ± 0.16	3.42 ± 0.14	3.23 ± 0.14
Bed Nucleus of Stria Terminalis	1.27 ± 0.05	1.25 ± 0.05	1.24 ± 0.06	1.17 ± 0.06
Hippocampus	19.9 ± 1.0	20.2 ± 1.0	20.1 ± 0.5	19.0 ± 0.9
Dentate Gyrus of Hippocampus	3.57 ± 0.18	3.60 ± 0.20	3.55 ± 0.09	3.37 ± 0.17
Basal Forebrain	4.22 ± 0.21	4.14 ± 0.18	4.16 ± 0.17	3.97 ± 0.16
Medial Septum	1.00 ± 0.09	0.97 ± 0.07	0.97 ± 0.06	0.92 ± 0.05
Stratum Granulosum of Hippocampus	0.84 ± 0.04	0.88 ± 0.07	0.85 ± 0.04	0.82 ± 0.06
Hypothalamus	10.0 ± 0.4	9.83 ± 0.40	9.71 ± 0.47	9.44 ± 0.52
Amygdala	12.2 ± 0.5	12.4 ± 1.0	12.2 ± 0.4	11.8 ± 0.7
Lateral Septum	3.13 ± 0.20	3.09 ± 0.18	3.02 ± 0.14	3.02 ± 0.22
Globus Pallidus	2.52 ± 0.13	2.50 ± 0.13	2.46 ± 0.18	2.39 ± 0.17
Mammillary Bodies	0.50 ± 0.05	0.50 ± 0.03	0.50 ± 0.05	0.50 ± 0.04
Fundus of Striatum	0.14 ± 0.03	0.14 ± 0.05	0.13 ± 0.03	0.13 ± 0.02

Supplementary Table S5. (Continued) Uncorrected volumes of brain structures at long-term EAE disease duration.

Anatomical Regions	Volume, mean ± SD mm^3			
	Naïve	CFA	Low Score EAE	High Score EAE
**Ventricles**				
Cerebral Aqueduct	0.46 ± 0.06	0.50 ± 0.05	0.47 ± 0.04	0.44 ± 0.05
Third Ventricle	1.12 ± 0.08	1.13 ± 0.10	1.07 ± 0.07	1.07 ± 0.10
Fourth Ventricle	0.42 ± 0.07	0.40 ± 0.05	0.45 ± 0.04	0.45 ± 0.09
Lateral Ventricle	3.77 ± 0.26	3.71 ± 0.19	3.75 ± 0.20	3.69 ± 0.37
**Cerebral Cortex**				
Parieto-Temporal Lobe	72.5 ± 2.3	72.4 ± 2.7	72.2 ± 2.2	66.8 ± 3.9
Frontal Lobe	40.6 ± 1.6	40.4 ± 1.2	40.0 ± 1.2	37.8 ± 1.8
Entorhinal Cortex	10.9 ± 0.4	11.0 ± 0.5	11.00 ± 0.5	10.1 ± 0.6
Occipital Lobe	6.09 ± 0.28	6.07 ± 0.24	5.93 ± 0.21	5.55 ± 0.34
**Brain Stem**				
Colliculus: Inferior	5.33 ± 0.29	5.37 ± 0.23	5.22 ± 0.12	4.84 ± 0.31
Colliculus: Superior	7.91 ± 0.49	7.94 ± 0.30	7.83 ± 0.22	7.21 ± 0.37
Midbrain	11.9 ± 0.7	12.1 ± 0.4	12.2 ± 0.5	11.5 ± 0.6
Ventral Tegmental Decussation	0.11 ± 0.01	0.11 ± 0.01	0.10 ± 0.01	0.10 ± 0.01
Inferior Olivary Complex	0.30 ± 0.05	0.28 ± 0.05	0.28 ± 0.03	0.29 ± 0.04
Facial Nerve (Cranial Nerve 7)	0.21 ± 0.3	0.21 ± 0.2	0.21 ± 0.2	0.22 ± 0.2
Corticospinal Tract/Pyramids	1.21 ± 0.20	1.16 ± 0.18	1.24 ± 0.14	1.25 ± 0.15
Pontine Nucleus	0.66 ± 0.07	0.64 ± 0.04	0.65 ± 0.06	0.65 ± 0.07
Superior Olivary Complex	0.62 ± 0.08	0.62 ± 0.05	0.60 ± 0.05	0.61 ± 0.06
Pons	14.5 ± 1.0	14.5 ± 0.5	14.7 ± 0.5	14.3 ± 0.9
Medulla	22.6 ± 1.8	22.0 ± 2.0	22.8 ±1.1	22.9 ± 2.1
Medial Lemniscus/Medial Longitudinal Fasciculus	2.2 ± 0.19	2.2 ± 0.17	2.2 ± 0.13	2.2 ± 0.17
Interpeduncular Nucleus	0.25 ± 0.03	0.26 ± 0.02	0.26 ± 0.02	0.25 ± 0.02
Cuneate Nucleus	0.26 ± 0.03	0.26 ± 0.03	0.26 ± 0.03	0.25 ± 0.04
**Cerebellum**				
Arbor Vita of Cerebellum	8.90 ± 0.29	9.00 ± 0.41	8.81 ± 0.29	8.16 ± 0.27
Cerebellar Cortex	45.0 ± 2.1	45.7 ± 2.3	45.3 ± 1.6	42.4 ± 2.3
Cerebellar Peduncle: Inferior	0.73 ± 0.04	0.71 ± 0.06	0.75 ± 0.03	0.72 ± 0.05
Cerebellar Peduncle: Middle	1.08 ± 0.07	1.06 ± 0.06	1.06 ± 0.05	1.03 ± 0.08
Cerebellar Peduncle: Superior	0.90 ± 0.07	0.90 ± 0.04	0.91 ± 0.07	0.90 ± 0.06
**Olfactory**				
Olfactory Tubercle	3.33 ± 0.20	3.38 ± 0.22	3.37 ± 0.24	3.16 ± 0.17
Lateral Olfactory Tract	1.24 ± 0.08	1.26 ± 0.06	1.24 ± 0.06	1.18 ± 0.07
Subependymale Zone / Rhinocele	0.06 ± 0.01	0.06 ± 0.01	0.06 ± 0.01	0.06 ± 0.01
Olfactory Bulbs	24.4 ± 2.5	23.2 ± 2.1	22.7 ± 1.3	22.7 ± 1.2

Supplementary Table S5. (Continued) Uncorrected volumes of brain structures at long-term EAE disease duration.

	Volume, mean ± SD mm^3			
Summed Regions	Naïve	CFA	Low Score EAE	High Score EAE
Cerebral Cortex	130 ± 4	130 ± 4	129 ± 4	120 ± 6
Cerebral Grey	229 ± 8	230 ± 7	228 ± 6	215 ± 10
Total Brain Volume	427 ± 15	423 ± 13	422 ± 9	399 ± 16
Cerebral White	30.6 ± 0.8	31.1 ± 0.8	30.9 ± 0.9	29.1 ± 1.3
Cerebellum	57.2 ± 2.4	57.0 ± 3.2	56.8 ± 1.9	53.2 ± 2.7
Olfactory	29.0 ± 2.6	27.9 ± 2.1	27.4 ± 1.4	27.1 ± 1.3
Brain Stem	71.5 ± 3.9	71.4 ± 3.1	72.3 ± 1.8	70.1 ± 3.8
Ventricles	5.80 ± 0.33	5.74 ± 0.30	5.74 ± 0.20	5.65 ± 0.43

Volumes mean ± standard deviation. EAE mice divided into above (High Score) and below (Low Score) average cumulative long-term disease scores.

Supplementary Table S6. Volumes of brain structures corrected for body weight at long-term EAE disease duration.

Anatomical Regions	Volume, mean ± SD mm^3			
	Naïve	CFA	Low Score EAE	High Score EAE
**White Matter**				
Corpus Callosum	17.3 ± 0.61	17.5 ± 0.50	17.3 ± 0.48	16.2 ± 0.88
Stria Terminalis	0.81 ± 0.03	0.85 ± 0.05	0.82 ± 0.06	0.77 ± 0.05
Optic Tract	1.47 ± 0.06	1.51 ± 0.04	1.46 ± 0.06	1.40 ± 0.07
Anterior Commissure: Pars Anterior	1.28 ± 0.06	1.28 ± 0.06	1.27 ± 0.08	1.21 ± 0.05
Internal Capsule	2.43 ± 0.11	2.45 ± 0.12	2.38 ± 0.20	2.30 ± 0.15
Stria Medullaris	0.72 ± 0.04	0.70 ± 0.04	0.71 ± 0.05	0.67 ± 0.04
Fasciculus Retroflexus	0.24 ± 0.02	0.25 ± 0.02	0.25 ± 0.01	0.23 ± 0.02
Fimbria	3.12 ± 0.16	3.18 ± 0.18	3.20 ± 0.10	3.07 ± 0.21
Anterior Commissure: Pars Posterior	0.41 ± 0.03	0.39 ± 0.04	0.39 ± 0.04	0.37 ± 0.04
Fornix	0.63 ± 0.02	0.63 ± 0.03	0.62 ± 0.04	0.61 ± 0.03
Habenular Commissure	0.03 ± 0.01	0.03 ± 0.01	0.03 ± 0.01	0.03 ± 0.01
Posterior Commissure	0.12 ± 0.01	0.12 ± 0.01	0.13 ± 0.01	0.12 ± 0.02
Mammilothalamic Tract	0.23 ± 0.01	0.23 ± 0.01	0.23 ± 0.01	0.23 ± 0.01
Cerebral Peduncle	2.03 ± 0.09	2.02 ± 0.09	2.02 ± 0.07	2.00 ± 0.14
**Grey Matter**				
Striatum	18.5 ± 0.55	18.4 ± 0.74	18.5 ± 0.62	17.2 ± 0.76
Thalamus	16.1 ± 0.27	16.3 ± 0.71	16.2 ± 0.47	15.4 ± 0.51
Pre-Para Subiculum	2.26 ± 0.10	2.23 ± 0.11	2.21 ± 0.09	2.06 ± 0.11
Periaqueductal Grey	3.66 ± 0.16	3.82 ± 0.25	3.73 ± 0.09	3.49 ± 0.24
Nucleus Accumbens	3.47 ± 0.16	3.44 ± 0.17	3.42 ± 0.14	3.22 ± 0.14
Hippocampus	20.1 ± 1.02	20.2 ± 1.02	20.0 ± 0.54	19.0 ± 0.86
Bed Nucleus of Stria Terminalis	1.26 ± 0.05	1.25 ± 0.05	1.23 ± 0.06	1.17 ± 0.06
Dentate Gyrus of Hippocampus	3.56 ± 0.20	3.60 ± 0.18	3.55 ± 0.09	3.37 ± 0.17
Basal Forebrain	4.21 ± 0.18	4.14 ± 0.21	4.16 ± 0.17	3.96 ± 0.16
Medial Septum	1.00 ± 0.07	0.97 ± 0.09	0.97 ± 0.06	0.91 ± 0.05
Stratum Granulosum of Hippocampus	0.85 ± 0.07	0.88 ± 0.04	0.85 ± 0.04	0.82 ± 0.06
Hypothalamus	10.0 ± 0.40	9.83 ± 0.44	9.69 ± 0.47	9.45 ± 0.52
Amygdala	12.4 ± 1.04	12.4 ± 0.50	12.3 ± 0.42	11.9 ± 0.72
Lateral Septum	3.12 ± 0.18	3.09 ± 0.20	3.02 ± 0.14	3.03 ± 0.22
Globus Pallidus	2.51 ± 0.13	2.50 ± 0.13	2.46 ± 0.18	2.38 ± 0.17
Mammillary Bodies	0.51 ± 0.03	0.50 ± 0.05	0.50 ± 0.05	0.49 ± 0.04
Fundus of Striatum	0.13 ± 0.03	0.13 ± 0.05	0.13 ± 0.03	0.13 ± 0.02

Supplementary Table S6. (Continued) Volumes of brain structures corrected for body weight at long-term EAE disease duration.

	Volume, mean ± SD mm^3			
Anatomical Regions	Naïve	CFA	Low Score EAE	High Score EAE
**Ventricles**				
Cerebral Aqueduct	0.47 ± 0.05	0.50 ± 0.06	0.47 ± 0.04	0.44 ± 0.05
Third Ventricle	1.12 ± 0.10	1.13 ± 0.08	1.08 ± 0.07	1.06 ± 0.10
Fourth Ventricle	0.43 ± 0.05	0.40 ± 0.07	0.44 ± 0.04	0.46 ± 0.09
Lateral Ventricle	3.74 ± 0.19	3.70 ± 0.26	3.74 ± 0.20	3.72 ± 0.37
**Cerebral Cortex**				
Parieto-Temporal Lobe	72.9 ± 2.7	72.3 ± 2.3	72.3 ± 2.2	66.6 ± 3.9
Frontal Lobe	40.9 ± 1.2	40.4 ± 1.6	40.1 ± 1.2	37.7 ± 1.8
Entorhinal Cortex	11.1 ± 0.5	11.0 ± 0.4	11.0 ± 0.5	10.1 ± 0.6
Occipital Lobe	6.07 ± 0.24	6.07 ± 0.28	5.94 ± 0.21	5.53 ± 0.34
**Brain Stem**				
Colliculus: Inferior	5.32 ± 0.23	5.37 ± 0.29	5.24 ± 0.12	4.80 ± 0.31
Colliculus: Superior	7.88 ± 0.30	7.93 ± 0.49	7.82 ± 0.22	7.21 ± 0.37
Midbrain	12.1 ± 0.4	12.1 ± 0.7	12.1 ± 0.5	11.5 ± 0.6
Ventral Tegmental Decussation	0.11 ± 0.01	0.11 ± 0.01	0.10 ± 0.01	0.11 ± 0.01
Inferior Olivary Complex	0.29 ± 0.05	0.28 ± 0.05	0.28 ± 0.03	0.29 ± 0.04
Facial Nerve (Cranial Nerve 7)	0.21 ± 0.2	0.21 ± 0.3	0.21 ± 0.2	0.22 ± 0.2
Corticospinal Tract/Pyramids	1.20 ± 0.18	1.15 ± 0.20	1.23 ± 0.14	1.27 ± 0.15
Pontine Nucleus	0.67 ± 0.04	0.64 ± 0.07	0.64 ± 0.06	0.66 ± 0.07
Superior Olivary Complex	0.63 ± 0.05	0.62 ± 0.08	0.60 ± 0.05	0.62 ± 0.06
Pons	14.6 ± 0.5	14.5 ± 1.0	14.7 ± 0.5	14.4 ± 0.9
Medulla	22.6 ± 2.0	22.0 ± 1.8	22.8 ±1.1	23.0 ± 2.1
Medial Lemniscus/Medial Longitudinal Fasciculus	2.15 ± 0.17	2.23 ± 0.19	2.19 ± 0.13	2.19 ± 0.17
Interpeduncular Nucleus	0.25 ± 0.02	0.26 ± 0.03	0.26 ± 0.02	0.25 ± 0.02
Cuneate Nucleus	0.26 ± 0.03	0.26 ± 0.03	0.26 ± 0.03	0.25 ± 0.04
**Cerebellum**				
Arbor Vita of Cerebellum	8.89 ± 0.41	9.00 ± 0.29	8.79 ± 0.29	8.14 ± 0.27
Cerebellar Cortex	45.4 ± 2.3	45.7 ± 2.1	45.4 ± 1.6	42.2 ± 2.3
Cerebellar Peduncle: Inferior	0.73 ± 0.06	0.71 ± 0.04	0.75 ± 0.03	0.71 ± 0.05
Cerebellar Peduncle: Middle	1.07 ± 0.06	1.06 ± 0.07	1.06 ± 0.05	1.03 ± 0.08
Cerebellar Peduncle: Superior	0.90 ± 0.04	0.90 ± 0.07	0.91 ± 0.07	0.90 ± 0.06
**Olfactory**				
Olfactory Tubercle	3.33 ± 0.22	3.38 ± 0.20	3.37 ± 0.24	3.15 ± 0.17
Lateral Olfactory Tract	1.26 ± 0.06	1.26 ± 0.08	1.24 ± 0.06	1.18 ± 0.07
Subependymale Zone / Rhinocele	0.06 ± 0.01	0.07 ± 0.01	0.06 ± 0.01	0.06 ± 0.01
Olfactory Bulbs	24.5 ± 2.09	23.2 ± 2.49	22.8 ± 1.27	22.8 ± 1.17

Supplementary Table S6. (Continued) Volumes of brain structures corrected for body weight at long-term EAE disease duration.

	Volume, mean ± SD mm^3			
Summed Regions	CFA	Naïve	Low Score EAE	High Score EAE
Cerebral Cortex	130 ± 4	131 ± 4	129 ± 4	119 ± 6
Cerebral Grey	230 ± 7	231 ± 8	228 ± 6	214 ± 10
Total Brain Volume	423 ± 13	426 ± 15	421 ± 9	399 ± 16
Cerebral White	31.1 ± 0.8	30.8 ± 0.8	30.8 ± 0.9	29.1 ± 1.3
Cerebellum	56.9 ± 3.2	57.1 ± 2.4	56.9 ± 1.9	53.0 ± 2.7
Olfactory	27.9 ± 2.1	29.1 ± 2.6	27.4 ± 1.4	27.1 ± 1.3
Brain Stem	71.4 ± 3.1	72.0 ± 3.9	72.1 ± 1.8	70.2 ± 3.8
Ventricles	5.74 ± 0.30	5.76 ± 0.33	5.73 ± 0.20	5.68 ± 0.43

Volumes mean ± standard deviation corrected for body weight using an ANCOVA analysis. EAE mice divided into above (High Score) and below (Low Score) average cumulative long-term disease scores.

Supplementary Table S7. Comparing the uncorrected volumes of Naïve, CFA, and Low Score EAE mice to High Score EAE mice.

	% Difference in Volume Versus High Score EAE					
Anatomical Region	Naïve	*q*	CFA	*q*	Low Score EAE	*q*
**White Matter**						
Corpus Callosum	-7.8	0.001	-8.4	1.2E-04	-7.0	0.001
Stria Terminalis	-6.0	0.095	-9.7	0.001	-6.1	0.039
Optic Tract	-5.5	0.051	-6.4	0.009	-5.7	0.015
Anterior Commissure: Pars Anterior	-7.9	0.014	-7.7	0.006	-4.0	0.181
Internal Capsule	-7.5	0.058	-8.1	0.014	-4.0	0.288
Stria Medullaris	-8.2	0.031	-5.8	0.106	-5.0	0.153
Fasciculus Retroflexus	-5.9	0.195	-7.1	0.053	-6.0	0.093
Fimbria	-3.6	0.402	-5.0	0.113	-4.5	0.134
Anterior Commissure: Pars Posterior	-10.4	0.086	-6.7	0.261	-5.6	0.342
Fornix	-3.4	0.336	-4.1	0.134	-1.0	0.829
Habenular Commissure	6.7	0.811	-10.6	0.442	-2.5	0.906
Posterior Commissure	-3.1	0.835	-3.9	0.733	-7.7	0.262
Mammilothalamic Tract	-3.2	0.338	-2.3	0.454	-0.5	0.906
Cerebral Peduncle	-2.3	0.988	-1.9	0.918	-0.5	0.994
**Grey Matter**						
Striatum	-7.9	4.2E-04	-7.4	2.0E-04	-6.5	4.4E-04
Thalamus	-5.0	0.009	-6.0	3.5E-04	-5.1	0.001
Pre-Para Subiculum	-8.7	0.002	-7.5	0.002	-6.9	0.003
Periaqueductal Grey	-5.5	0.118	-8.9	0.001	-7.2	0.006
Nucleus Accumbens	-8.5	0.001	-7.4	0.001	-5.4	0.015
Bed Nucleus of Stria Terminalis	-8.8	0.001	-7.4	0.002	-4.5	0.061
Hippocampus	-6.5	0.014	-6.2	0.007	-5.8	0.008
Dentate Gyrus of Hippocampus	-6.1	0.035	-6.8	0.006	-5.8	0.014
Basal Forebrain	-6.6	0.012	-4.9	0.038	-4.8	0.032
Medial Septum	-10.5	0.009	-6.8	0.071	-5.1	0.182
Stratum Granulosum of Hippocampus	-5.2	0.297	-8.1	0.025	-4.1	0.330
Hypothalamus	-6.3	0.035	-4.6	0.093	-2.3	0.474
Amygdala	-6.3	0.101	-5.8	0.081	-4.4	0.197
Lateral Septum	-5.6	0.149	-4.2	0.248	1.2	0.839
Globus Pallidus	-5.5	0.196	-4.8	0.201	-3.7	0.329
Mammillary Bodies	-4.5	0.549	-3.1	0.717	-3.3	0.603
Fundus of Striatum	0.5	0.990	0.7	0.988	-7.9	0.599

Supplementary Table S7. (Continued) Comparing the uncorrected volumes of Naïve, CFA, and Low Score EAE mice to High Score EAE mice.

	% Difference in Volume Versus High Score EAE					
Anatomical Region	Naïve	*q*	CFA	*q*	Low Score EAE	*q*
**Ventricles**						
Cerebral Aqueduct	-5.9	0.468	-11.2	0.033	-6.9	0.232
Third Ventricle	-6.2	0.286	-6.7	0.161	-1.7	0.836
Fourth Ventricle	5.3	0.751	11.9	0.234	4.6	0.739
Lateral Ventricle	-3.1	0.644	-1.3	0.870	-1.4	0.833
**Cerebral Cortex**						
Parieto-Temporal Lobe	-9.4	1.13E-04	-8.3	1.17E-04	-8.6	5.36E-05
Frontal Lobe	-8.3	1.77E-04	-6.9	3.49E-04	-6.4	4.50E-04
Entorhinal Cortex	-9.7	4.07E-04	-8.2	0.001	-8.5	2.12E-04
Occipital Lobe	-9.4	0.001	-9.1	1.83E-04	-7.6	0.001
**Brain Stem**						
Colliculus: Inferior	-8.2	1.16E-03	-9.2	9.64E-05	-9.6	2.66E-05
Colliculus: Superior	-8.2	1.29E-03	-8.6	1.89E-04	-9.0	7.50E-05
Midbrain	-4.7	0.095	-5.3	0.022	-4.7	0.035
Ventral Tegmental Decussation	-7.8	0.195	-3.9	0.572	2.9	0.741
Inferior Olivary Complex	-4.7	0.777	-0.1	1.000	1.5	0.918
Facial Nerve (Cranial Nerve 7)	3.7	0.721	6.6	0.296	3.7	0.598
Corticospinal Tract/Pyramids	3.6	0.823	8.2	0.401	2.4	0.854
Pontine Nucleus	-2.4	0.823	2.9	0.751	0.7	0.934
Superior Olivary Complex	-3.3	0.751	-1.3	0.897	2.9	0.734
Pons	-2.5	0.591	-1.6	0.750	-1.9	0.612
Medulla	0.2	0.988	3.4	0.602	0.2	0.992
Medial Lemniscus/Medial Longitudinal Fasciculus	-1.5	0.868	1.8	0.816	0.02	1.000
Interpeduncular Nucleus	-3.2	0.762	-3.8	0.602	-0.6	0.944
Cuneate Nucleus	0.3	0.995	-1.4	0.918	-2.0	0.876
**Cerebellum**						
Arbor Vita of Cerebellum	-8.6	2.19E-04	-9.6	2.02E-05	-7.6	1.08E-04
Cerebellar Cortex	-7.5	0.006	-7.9	0.001	-7.5	0.001
CBL Peduncle: Inferior	-2.0	0.789	1.5	0.829	-6.0	0.072
CBL Peduncle: Middle	-5.4	0.221	-4.1	0.317	-2.6	0.585
CBL Peduncle: Superior	-1.7	0.836	-1.4	0.840	-0.8	0.899
**Olfactory**						
Olfactory Tubercle	-7.0	0.075	-8.3	0.011	-5.9	0.069
Lateral Olfactory Tract	-7.3	0.021	-8.2	0.002	-4.1	0.155
Subependymale Zone / Rhinocele	-11.7	0.080	-12.6	0.024	2.8	0.804
Olfactory Bulbs	-6.7	0.287	-1.45	0.700	0.9	0.953

Supplementary Table S7. (Continued) Comparing the uncorrected volumes of Naïve, CFA, and Low Score EAE mice to High Score EAE mice.

	% Difference in Volume Versus High Score EAE					
Summed Regions	Naïve	*q*	CFA	*q*	Low Score EAE	*q*
Cerebral Cortex	-9.1	8.2E-05	-7.9	9.0E-05	-7.9	4.9E-05
Cerebral Grey	-8.0	1.1E-04	-7.2	1.1E-04	-6.6	1.1E-04
Total Brain Volume	-7.1	2.4E-04	-6.1	2.8E-04	-5.8	2.7E-04
Cerebral White	-6.6	0.001	-7.3	1.00E-04	-5.6	0.001
Cerebellum	-7.5	0.006	-7.2	0.002	-7.3	0.001
Olfactory	-6.5	0.237	-2.3	0.342	-0.1	0.842
Brain Stem	-3.1	0.369	-2.1	0.548	-3.1	0.238
Ventricles	-3.3	0.496	-2.3	0.623	-1.5	0.795

% volume difference compares average volume between High Score EAE and other groups (positive indicates increase in size, negative indicates decrease in size). No significant differences in volume were detected between Naïve, CFA, and low score EAE groups. q-values (significance = <0.1) represent FDR-adjusted p-values.

Supplementary Table S8. Comparing the volumes, corrected for body weight, of Naïve, CFA, and Low Score EAE mice to High Score EAE mice.

Anatomical Region	% Difference in Volume Versus High Score EAE					
	Naïve	*q*	CFA	*q*	Low Score EAE	*q*
**White Matter**						
Corpus Callosum	-7.5	0.003	-6.5	9.5E-05	-6.7	3.5E-04
Stria Terminalis	-9.2	0.074	-5.2	0.001	-6.2	0.029
Optic Tract	-7.0	0.062	-5.0	0.001	-4.3	0.059
Internal Capsule	-7.1	0.107	-6.3	0.023	-4.4	0.230
Anterior Commissure: Pars Anterior	-6.0	0.099	-5.8	0.033	-4.7	0.128
Fasciculus Retroflexus	-5.8	0.369	-4.1	0.089	-5.6	0.104
Stria Medullaris	-4.2	0.092	-6.1	0.222	-4.6	0.164
Fimbria	-3.6	0.539	-1.7	0.223	-4.2	0.140
Anterior Commissure: Pars Posterior	-5.6	0.123	-8.7	0.310	-4.7	0.436
Habenular Commissure	-11.1	0.755	5.6	0.359	-3.1	0.777
Fornix	-3.4	0.481	-2.6	0.213	-1.7	0.630
Posterior Commissure	-3.2	0.794	-1.7	0.709	-6.5	0.359
Mammilothalamic Tract	-0.8	0.694	-1.5	0.763	-1.7	0.619
Cerebral Peduncle	-1.1	0.731	-1.5	0.758	-1.1	0.752
**Grey Matter**						
Striatum	-6.6	0.001	-6.7	1.3E-04	-6.2	2.7E-04
Thalamus	-5.3	0.020	-4.1	2.6E-04	-4.8	0.001
Pre-Para Subiculum	-7.6	0.001	-8.7	0.001	-6.5	0.004
Periaqueductal Grey	-8.6	0.132	-4.8	4.0E-04	-6.4	0.010
Nucleus Accumbens	-6.2	0.004	-7.2	0.004	-5.9	0.007
Hippocampus	-5.6	0.016	-5.4	0.003	-4.9	0.011
Bed Nucleus of Stria Terminalis	-6.0	0.006	-7.1	0.008	-4.9	0.038
Dentate Gyrus of Hippocampus	-6.3	0.043	-5.2	0.003	-5.0	0.023
Basal Forebrain	-4.4	0.016	-5.9	0.047	-4.8	0.028
Medial Septum	-5.4	0.025	-9.0	0.180	-6.1	0.112
Hypothalamus	-4.0	0.050	-5.6	0.122	-2.6	0.394
Stratum Granulosum of Hippocampus	-6.4	0.630	-2.7	0.051	-3.6	0.369
Amygdala	-4.9	0.179	-5.1	0.115	-4.1	0.220
Globus Pallidus	-4.6	0.210	-5.1	0.180	-3.2	0.411
Lateral Septum	-1.8	0.570	-2.8	0.686	0.5	0.947
Mammillary Bodies	-3.1	0.529	-4.3	0.630	-2.5	0.695
Lateral Ventricle	0.4	0.966	-0.5	0.966	-0.6	0.949

Supplementary Table S8. (Continued) Comparing the volumes, corrected for body weight, of Naïve, CFA, and Low Score EAE mice to High Score EAE mice.

Anatomical Region	% Difference in Volume Versus High Score EAE					
	Naïve	*q*	CFA	*q*	Low Score EAE	*q*
**Ventricles**						
Cerebral Aqueduct	-11.7	0.386	-6.2	0.012	-5.6	0.366
Third Ventricle	-5.8	0.387	-5.0	0.197	-1.3	0.773
Fourth Ventricle	13.2	0.618	6.5	0.139	3.8	0.737
Lateral Ventricle	0.4	0.966	-0.5	0.966	-0.6	0.949
**Cerebral Cortex**						
Parieto-Temporal Lobe	-8.0	4.9E-05	-8.7	3.0E-05	-7.9	3.3E-05
Frontal Lobe	-6.8	9.7E-05	-8.0	1.3E-04	-6.0	0.001
Entorhinal Cortex	-7.5	2.5E-04	-8.5	2.2E-04	-7.7	1.5E-04
Occipital Lobe	-8.8	2.5E-04	-8.8	5.3E-05	-6.8	0.001
**Brain Stem**						
Colliculus: Inferior	-10.5	1.2E-04	-9.7	1.7E-05	-8.3	1.4E-04
Colliculus: Superior	-9.1	2.5E-04	-8.5	2.5E-05	-7.8	1.4E-04
Midbrain	-4.4	0.037	-4.9	0.028	-4.8	0.015
Ventral Tegmental Decussation	-2.0	0.360	-5.9	0.756	1.2	0.792
Corticospinal Tract/Pyramids	9.7	0.629	5.7	0.241	2.8	0.752
Facial Nerve (Cranial Nerve 7)	6.5	0.653	3.7	0.250	3.8	0.573
Medulla	4.2	0.777	1.4	0.419	0.8	0.943
Pontine Nucleus	3.0	0.760	-1.8	0.660	2.0	0.747
Superior Olivary Complex	-0.2	0.763	-1.9	0.996	2.6	0.710
Medial Lemniscus/Medial Longitudinal Fasciculus	2.3	0.946	-0.7	0.685	0.7	0.808
Pons	-0.9	0.712	-1.6	0.762	-1.8	0.626
Inferior Olivary Complex	2.8	0.978	-0.8	0.762	2.6	0.763
Interpeduncular Nucleus	-2.3	0.771	-1.8	0.734	-2.4	0.734
Cuneate Nucleus	-3.6	0.773	-2.5	0.707	-1.2	0.946
**Cerebellum**						
Arbor Vita of Cerebellum	-9.6	1.0E-04	-8.5	8.9E-06	-7.4	9.3E-05
Cerebellar Cortex	-7.7	0.005	-7.0	4.7E-04	-7.0	0.001
CBL Peduncle: Inferior	0.8	0.645	-2.5	0.796	-5.0	0.146
CBL Peduncle: Middle	-2.8	0.406	-3.8	0.509	-2.8	0.516
CBL Peduncle: Superior	-0.5	0.958	-0.6	0.953	-1.0	0.775
**Olfactory**						
Olfactory Tubercle	-6.9	0.186	-5.4	0.028	-6.6	0.038
Lateral Olfactory Tract	-6.0	0.205	-4.7	0.029	-4.9	0.107
Subependymale Zone / Rhinocele	-9.0	0.321	-7.8	0.138	-0.7	0.974
Olfactory Bulbs	-2.2	0.102	-7.3	0.693	-0.3	0.975

Supplementary Table S8. (Continued) Comparing the volumes, corrected for body weight, of Naïve, CFA, and Low Score EAE mice to High Score EAE mice.

Summed Regions	% Difference in Volume Versus High Score EAE					
	Naïve	*q*	CFA	*q*	Low Score EAE	*q*
Cerebral Cortex	-7.6	2.2E-05	-8.5	3.0E-05	-7.3	3.2E-05
Cerebral Grey	-6.6	2.8E-05	-7.2	8.8E-05	-6.1	9.1E-05
Total Brain Volume	-5.7	1.2E-04	-6.3	1.7E-04	-5.4	2.2E-04
Cerebral White	-6.3	1.1E-04	-5.3	0.004	-5.4	0.001
Cerebellum Total	-6.9	0.001	-7.1	0.004	-6.9	0.001
Olfactory	-2.9	0.499	-6.9	0.070	-1.3	0.756
Brain Stem	-1.6	0.616	-2.3	0.485	-2.6	0.325
Ventricles	-0.9	0.763	-1.3	0.754	-0.8	0.773

% volume difference compares average volume between High Score EAE and other groups (positive indicates increase in size, negative indicates decrease in size). No significant differences in volume were detected between Naïve, CFA, and low score EAE groups. q-values (significance = <0.1) represent FDR-adjusted p-values.

Supplementary Table S9. Comparing the relative changes in volume from peak clinical (days 14-16) to long-term (66+) disease duration of Naïve, CFA, to EAE mice.

Anatomical Region	Percent change in volume; peak to long-term mean ± SD %			Post-hoc *q* values		
	Naïve	CFA	EAE	Naïve vs. CFA	Naïve vs. EAE	CFA vs. EAE
**White Matter**						
Fimbria	4.7 ± 5.51	3.29 ± 3.78	-4.22 ± 5.80	0.721	0.007	0.029
Corpus Callosum	4.0 ± 4.07	4.25 ± 4.09	-2.88 ± 5.66	0.987	0.016	0.025
Optic Tract	5.8 ± 2.67	7.36 ± 4.41	-0.61 ± 7.19	0.687	0.038	0.024
Cerebral Peduncle	-8.5 ± 5.22	6.48 ± 6.28	0.09 ± 6.50	0.631	0.016	0.096
Stria Medullaris	10.5 ± 9.61	-1.66 ± 5.96	-1.89 ± 7.96	0.026	0.010	1.000
Fornix	7.48 ± 8.09	0.59 ± 6.47	-4.16 ± 8.62	0.174	0.010	0.328
Anterior Commissure: Pars Anterior	7.45 ± 6.00	2.27 ± 3.67	-1.41 ± 6.45	0.202	0.015	0.268
Fasciculus Retroflexus	6.91 ± 7.81	0.89 ± 5.64	-2.54 ± 6.72	0.096	0.037	0.064
Internal Capsule	-9.47 ± 8.89	-4.55 ± 8.92	-1.97 ± 9.93	1	0.032	0.424
Anterior Commissure: Pars Posterior	14.63 ± 13.31	-2.09 ± 11.17	0.65 ± 15.96	0.471	0.177	0.499
Stria Terminalis	6.29 ± 6.62	5.99 ± 6.55	0.49 ± 6.35	0.268	0.205	0.834
Habenular Commissure	-2.60 ± 31.27	26.74 ± 40.56	-3.66 ± 24.81	0.735	0.230	0.169
Posterior Commissure	7.28 ± 12.85	2.56 ± 9.87	-2.55 ± 20.66	0.706	0.013	0.343
Mammilothalamic Tract	5.63 ± 7.11	2.22 ± 5.61	3.07 ± 7.45	0.484	0.532	0.878
**Grey Matter**						
Hippocampus	5.11 ± 2.36	3.11 ± 3.10	-3.35 ± 3.14	0.282	3.76E-05	0.001
Basal Forebrain	10.00 ± 4.26	1.86 ± 4.32	-3.93 ± 5.00	0.013	4.08E-05	0.049
Lateral Septum	12.95 ± 5.41	5.74 ± 1.13	-2.08 ± 7.70	0.085	3.63E-04	0.047
Periaqueductal Grey	3.35 ± 7.95	3.73 ± 3.79	-7.32 ± 5.86	0.976	0.006	0.010
Dentate Gyrus of Hippocampus	5.43 ± 1.62	3.17 ± 4.58	-0.25 ± 4.39	0.328	0.006	0.114
Nucleus Accumbens	7.52 ± 4.51	2.77 ± 2.75	-1.23 ± 5.94	0.156	0.004	0.205
Amygdala	8.95 ± 5.64	3.31 ± 3.07	0.69 ± 5.72	0.114	0.010	0.454
Pre-Para Subiculum	5.58 ± 3.58	1.49 ± 2.64	-0.71 ± 5.28	0.127	0.010	0.427
Medial Septum	16.55 ± 13.22	4.43 ± 4.93	2.36 ± 9.70	0.304	0.016	0.471
Bed Nucleus of Stria Terminalis	8.76 ± 6.36	-2.48 ± 5.80	-0.72 ± 7.34	0.084	0.019	0.13
Thalamus	4.14 ± 3.29	2.96 ± 3.30	-0.02 ± 3.50	0.169	0.022	0.784
Hypothalamus	10.79 ± 5.76	3.16 ± 4.02	1.85 ± 8.14	0.593	0.019	0.454
Striatum	4.00 ± 3.05	2.03 ± 3.25	-0.49 ± 4.72	0.151	0.084	0.783
Fundus of Striatum	36.81 ± 55.29	15.48 ± 10.17	9.74 ± 28.78	0.992	0.115	0.319
Globus Pallidus	8.86 ± 8.64	4.90 ± 5.96	1.96 ± 8.89	0.785	0.157	0.513
Stratum Granulosum of Hippocampus	1.92 ± 6.39	2.92 ± 3.42	-0.94 ± 6.19	0.706	0.691	0.454
Mammillary Bodies	10.56 ± 13.67	3.71 ± 2.98	5.93 ± 9.86	0.340	0.452	0.765

Supplementary Table S9. (Continued) Comparing the relative changes in volume from peak clinical (days 14-16) to long-term (66+) disease duration of Naïve, CFA, to EAE mice.

	Percent change in volume; peak to long-term mean ± SD %			Post-hoc *q* values		
Anatomical Region	Naïve	CFA	EAE	Naïve vs. CFA	Naïve vs. EAE	CFA vs. EAE
**Ventricles**						
Cerebral Aqueduct	-1.39 ± 8.12	13.17 ± 8.00	-11.08 ± 6.84	0.008	0.025	7.25E-05
Lateral Ventricle	8.48 ± 4.32	5.41 ± 5.50	-3.95 ± 6.53	0.442	0.001	0.014
Fourth Ventricle	8.68 ± 22.04	-17.92 ± 15.46	-16.38 ± 9.78	0.018	0.010	0.918
Third Ventricle	10.40 ± 9.18	10.40 ± 6.12	-0.22 ± 10.20	0.201	0.034	0.291
**Cerebral Cortex**						
Entorhinal Cortex	3.51 ± 3.39	3.39 ± 2.50	-1.19 ± 3.44	0.994	0.010	0.021
Parieto-Temporal Lobe	2.48 ± 1.90	0.83 ± 1.79	-1.65 ± 3.75	0.392	0.013	0.343
Occipital Lobe	3.58 ± 4.44	-1.02 ± 1.94	0.68 ± 4.08	0.084	0.068	0.694
Frontal Lobe	3.16 ± 3.55	2.48 ± 1.82	1.47 ± 3.14	0.600	0.23	0.543
**Brain Stem**						
Cuneate Nucleus	2.58 ± 12.03	10.21 ± 14.32	-13.6 ± 9.93	0.339	0.017	0.004
Midbrain	4.98 ± 4.27	2.00 ± 4.53	-2.57 ± 4.05	0.290	0.004	0.047
Colliculus: Superior	3.23 ± 6.57	3.26 ± 5.18	-4.89 ± 4.78	1.000	0.011	0.021
Colliculus: Inferior	1.65 ± 6.58	3.90 ± 3.42	-5.04 ± 5.74	0.576	0.037	0.017
Ventral Tegmental Decussation	22.57 ± 21.14	-11.89 ± 10.69	-6.71 ± 13.53	0.004	0.004	0.632
Pons	5.60 ± 5.50	0.83 ± 3.72	-3.64 ± 5.27	0.165	0.004	0.170
Medulla	4.72 ± 8.02	-0.53 ± 6.28	-4.54 ± 7.71	0.455	0.038	0.784
Superior Olivary Complex	12.44 ± 13.36	1.37 ± 11.07	-0.78 ± 9.83	0.314	0.042	0.319
Medial Lemniscus/Medial Longitudinal Fasciculus	8.53 ± 9.07	2.63 ± 5.28	0.51 ± 7.57	0.076	0.115	0.187
Interpeduncular Nucleus	7.79 ± 13.63	11.03 ± 22.47	-2.83 ± 12.81	0.165	0.138	0.595
Inferior Olivary Complex	12.85 ± 19.34	7.33 ± 9.67	0.96 ± 12.34	0.488	0.206	0.606
Facial Nerve (Cranial Nerve 7)	-5.52 ± 14.11	-3.76 ± 11.18	2.44 ± 12.44	0.828	0.294	0.63
Pontine Nucleus	8.33 ± 14.87	4.87 ± 11.31	0.70 ± 11.31	0.286	0.294	0.646
Corticospinal Tract/Pyramids	2.62 ± 14.00	1.54 ± 10.63	-1.38 ± 12.12	0.938	0.594	0.735
**Cerebellum**						
Arbor Vita of Cerebellum	3.58 ± 4.36	-0.21 ± 2.30	-8.19 ± 3.20	0.111	3.59E-05	0.002
Cerebellar Cortex	-0.17 ± 2.70	-0.14 ± 2.60	-6.13 ± 4.58	1.000	0.004	0.009
CBL Peduncle: Inferior	9.33 ± 8.19	8.21 ± 5.67	-1.46 ± 6.93	0.826	0.008	0.025
CBL Peduncle: Superior	4.90 ± 7.31	-5.40 ± 2.22	-5.33 ± 6.69	0.015	0.006	1.000
CBL Peduncle: Middle	8.93 ± 5.72	4.58 ± 6.23	0.14 ± 7.23	0.631	0.018	0.779

Supplementary Table S9. (Continued) Comparing the relative changes in volume from peak clinical (days 14-16) to long-term (66+) disease duration of Naïve, CFA, to EAE mice.

	Percent change in volume; peak to long-term mean ± SD %			Post-hoc q values		
Anatomical Region	Naïve	CFA	EAE	Naïve vs. CFA	Naïve vs. EAE	CFA vs. EAE
**Olfactory**						
Olfactory Tubercle	9.48 ± 4.86	3.20 ± 9.74	-4.96 ± 6.31	0.169	0.001	0.059
Lateral Olfactory Tract	7.39 ± 4.77	5.88 ± 5.01	-1.08 ± 4.09	0.632	0.003	0.018
Subependymale Zone / Rhinocele	9.06 ± 12.28	5.47 ± 12.73	-3.91 ± 17.57	0.419	0.991	0.126
Olfactory Bulbs	14.28 ± 12.08	11.61 ± 5.97	7.66 ± 9.13	0.607	0.396	0.306

Supplementary Table S9. (Continued) Comparing the relative changes in volume from peak clinical (days 14-16) to long-term (66+) disease duration of Naïve, CFA, to EAE mice.

	Percent change in volume; peak to long-term mean ± SD %			Post-hoc q values		
Anatomical Region	Naïve	CFA	EAE	Naïve vs. CFA	Naïve vs. EAE	CFA vs. EAE
Total Brain Volume	4.60 ± 1.01	2.32 ± 1.22	-1.90 ± 2.69	0.098	3.88E-05	0.004
Ventricles	7.91 ± 4.90	4.71 ± 4.54	-5.04 ± 5.29	0.354	2.03E-04	0.006
Cerebellum	0.72 ± 2.17	-0.06 ± 2.43	-6.29 ± 4.06	0.712	3.87E-04	0.003
Cerebral Grey	4.43 ± 1.36	2.09 ± 1.72	-0.69 ± 2.80	0.099	3.35E-04	0.038
Brain Stem	4.62 ± 3.80	1.39 ± 4.10	-4.09 ± 3.74	0.208	0.001	0.026
Cerebral White	5.33 ± 2.11	4.08 ± 4.31	-2.52 ± 4.52	0.650	0.002	0.011
Cerebral Cortex	2.94 ± 2.09	1.45 ± 1.19	-0.56 ± 3.14	0.369	0.014	0.191
Olfactory	13.34 ± 9.83	10.12 ± 4.92	5.58 ± 8.08	0.586	0.095	0.401

% change in volume compares average volumes at peak and long-term for each group (positive indicates increase in size, negative indicates decrease in size). q-values (significance = <0.1) represent FDR-adjusted p-values.

Supplementary Table S10. Correlations of long-term brain regional volumes and cumulative behavior scores in EAE mice.

Anatomical Regions	*q*	R^2
CTX: Entorhinal Cortex	3.5E-04	0.59
CTX: Parieto-Temporal Lobe	1.6E-04	0.57
Colliculus: Superior	0.001	0.50
Cerebral Cortex: Frontal Lobe	0.001	0.49
Striatum	0.001	0.48
Hippocampus	0.001	0.46
Thalamus	0.001	0.46
Corpus Callosum	0.001	0.45
Arbor Vita of Cerebellum	0.002	0.41
Periaqueductal Grey	0.003	0.39
Dentate Gyrus of Hippocampus	0.004	0.38
Cerebellar Cortex	0.004	0.38
Cerebral Cortex: Occipital Lobe	0.007	0.34
Colliculus: Inferior	0.008	0.33
Bed Nucleus of Stria Terminalis	0.008	0.33
Pre-Para Subiculum	0.009	0.32
Olfactory Tubercle	0.010	0.31
Midbrain	0.013	0.29
Amygdala	0.015	0.29
Lateral Olfactory Tract	0.016	0.28
Basal Forebrain	0.018	0.27
Stria Medullaris	0.018	0.27
Nucleus Accumbens	0.018	0.26
Anterior Commissure: Pars Anterior	0.020	0.26
Fasciculus Retroflexus	0.024	0.25
Fimbria	0.038	0.22
Medial Septum	0.113	0.18
Cerebellar Peduncle: Inferior	0.124	0.17
Optic Tract	0.130	0.16
Stria Terminalis	0.137	0.15
Cerebral Aqueduct	0.202	0.11
Cerebellar Peduncle: Superior	0.267	0.09
Anterior Commissure: Pars Posterior	0.293	0.08
Internal Capsule	0.338	0.07
Stratum Granulosum of Hippocampus	0.362	0.07
Interpeduncular Nucleus	0.356	0.07
Cerebellar Peduncle: Middle	0.366	0.06

Supplementary Table S10. Correlations of long-term brain regional volumes and cumulative behavior scores in EAE mice.

Anatomical Regions	*q*	R^2
Facial Nerve (Cranial Nerve 7)	0.380	0.06
Posterior Commissure	0.408	0.05
Corticospinal Tract/Pyramids	0.519	0.04
Pontine Nucleus	0.520	0.04
Pons	0.525	0.04
Hypothalamus	0.536	0.03
Lateral Ventricle	0.541	0.03
Subependymale Zone /Rhinocele	0.566	0.03
Medulla	0.560	0.03
Mammilothalamic Tract	0.657	0.02
Ventral Tegmental Decussation	0.695	0.02
Lateral Septum	0.713	0.01
Habenular Commissure	0.744	0.01
Fornix	0.766	0.01
Inferior Olivary Complex	0.770	0.01
Fundus of Striatum	0.786	0.009
Globus Pallidus	0.805	0.008
Superior Olivary Complex	0.800	0.007
Medial Lemniscus/Longitudinal Fasciculus	0.827	0.006
Mammillary Bodies	0.860	0.005
Olfactory Bulbs	0.935	0.003
Fourth Ventricle	0.933	0.003
Cerebral Peduncle	0.959	0.002
Third Ventricle	0.962	0.002
Cuneate Nucleus	0.976	0.002

Summed Regions	*q*	R^2
Cerebral Cortex	1.75E-04	0.59
Cerebral Grey	1.40E-04	0.57
Total Brain Volume	0.001	0.52
Cerebral White	0.002	0.41
Cerebellum	0.003	0.40
Ventricles	0.500	0.04
Olfactory	0.592	0.03
Brain Stem	0.637	0.06

*q values were corrected for multiple comparisons using the false discovery rate (FDR) method. A 10% threshold was used for significance. Regressions were controlled for bodyweight.

Supplementary Table S11. Correlations of relative change in brain volume from peak to long-term and cumulative behavior scores in EAE mice.

Anatomical Regions	*q*	R^2
Colliculus: Superior	0.003	0.61
Cerebral Cortex: Entorhinal Cortex	0.006	0.55
Cerebral Cortex: Parieto-Temporal Lobe	0.007	0.54
Cerebral Cortex: Occipital Lobe	0.012	0.48
Mammilothalamic Tract	0.026	0.40
Corpus Callosum	0.028	0.40
Cerebral Peduncle	0.030	0.39
Periaqueductal Grey	0.031	0.39
Dentate Gyrus of Hippocampus	0.031	0.39
Hippocampus	0.036	0.37
Globus Pallidus	0.048	0.34
Facial Nerve (Cranial Nerve 7)	0.049	0.33
Medial Lemniscus/Medial Longitudinal Fasciculus	0.051	0.33
Medulla	0.068	0.30
Fourth Ventricle	0.106	0.24
Cerebellar Cortex	0.109	0.24
Fimbria	0.112	0.23
Hypothalamus	0.132	0.22
Pontine Nucleus	0.132	0.21
Mammillary Bodies	0.138	0.21
Colliculus: Inferior	0.139	0.21
Fornix	0.215	0.15
Arbor Vita of Cerebellum	0.217	0.15
Thalamus	0.235	0.14
Cerebral Cortex: Frontal Lobe	0.243	0.13
Cerebellar Peduncle: Superior	0.248	0.13
Optic Tract	0.260	0.12
Cerebral Aqueduct	0.267	0.12
Cerebellar Peduncle: Middle	0.319	0.10
Third Ventricle	0.339	0.09
Stratum Granulosum of Hippocampus	0.350	0.09
Fundus of Striatum	0.351	0.09
Superior Olivary Complex	0.384	0.08
Inferior Olivary Complex	0.408	0.07
Subependymale Zone / Rhinocele	0.416	0.07
Basal Forebrain	0.423	0.07
Pons	0.426	0.06

Supplementary Table S11. Correlations of relative change in brain volume from peak to long-term and cumulative behavior scores in EAE mice.

Anatomical Regions	*q*	R^2
Nucleus Accumbens	0.434	0.06
Medial Septum	0.438	0.06
Striatum	0.444	0.06
Olfactory Bulbs	0.445	0.06
Ventral Tegmental Decussation	0.461	0.06
Midbrain	0.489	0.05
Stria Medullaris	0.492	0.05
Habenular Commissure	0.495	0.05
Corticospinal Tract/Pyramids	0.500	0.05
Anterior Commissure: Pars Posterior	0.502	0.05
Stria Terminalis	0.519	0.04
Lateral Septum	0.525	0.04
Pre-Para Subiculum	0.556	0.04
Amygdala	0.558	0.04
Lateral Olfactory Tract	0.573	0.03
Internal Capsule	0.589	0.03
Lateral Ventricle	0.647	0.02
Olfactory Tubercle	0.695	0.02
Interpeduncular Nucleus	0.735	0.01
Cerebellar Peduncle: Inferior	0.803	0.007
Anterior Commissure: Pars Anterior	0.832	0.005
Fasciculus Retroflexus	0.851	0.004
Bed Nucleus of Stria Terminalis	0.870	0.003
Posterior Commissure	0.914	0.001
Cuneate Nucleus	0.993	8.0E-06

Summed Regions	*q*	R^2
Cerebral Cortex	0.009	0.51
Cerebellum	0.109	0.24
Cerebral Grey	0.131	0.21
Cerebral White	0.238	0.14
Total Brain Volume	0.341	0.09
Brain Stem	0.407	0.07
Olfactory	0.512	0.04
Ventricles	0.901	0.002

*q values were corrected for multiple comparisons using the false discovery rate (FDR) method. A 10% threshold was used for significance.
